# Vision-Guided Robotic Arm with Deep Learning and Secure Digital Twin for Pre-Deployment Quadruped Robot Operation

**DOI:** 10.3390/s26217064

**Published:** 2026-11-08

**Authors:** Shih-Hao Chang, Minyechil Alehegn Tefera, Jun-Ming Cheng, Zhen-Cheng Li, Ching-Yuan Chang, Tzu-Hsiu Chang, Tien-Fu Lu, Pai-Yen Chen, Chin-Sheng Chen

**Affiliations:** 1 Department of Computer Science and Information Engineering, National Taipei University of Technology, Taipei 10608, Taiwan; sh.chang@ntut.edu.tw; 2 Department of Mechanical Engineering, National Taipei University of Technology, Taipei 10608, Taiwan; minyechil.alehegn@ntut.edu.tw (M.A.T.); t112568049@ntut.edu.tw (J.-M.C.); yah205318@gmail.com (Z.-C.L.); chang@ntut.edu.tw (C.-Y.C.); rex.chang@cloudcap.cc (T.-H.C.); 3 YCL Technology Inc., Taipei 104090, Taiwan; 4 School of Electrical and Mechanical Engineering, Adelaide University, Adelaide, SA 5005, Australia; tien-fu.lu@adelaide.edu.au; 5 Department of Electrical and Computer Engineering, University of Illinois Chicago, Chicago, IL 60607, USA; pychen@uic.edu; 6 Graduate Institute of Automation Technology, National Taipei University of Technology, Taipei 10608, Taiwan

**Keywords:** artificial intelligence (AI), object detection, vision-guided robotic arm, quadruped robot, digital twin, YOLO-based detection, robotic arm, cyber–physical systems

## Abstract

In this article, we propose a vision-guided robotic arm system that integrates deep learning, digital twin, and cybersecurity to enhance the maintenance of quadruped robots. The proposed system comprises two core components. (1) A YOLOv11-based object detection system with depth cameras identifies maintenance targets and extracts 3D coordinates for autonomous robotic arm localization and manipulation. (2) A digital twin environment is used to simulate and validate path planning, trajectory stability, and operational safety before real-time deployment. Moreover, we propose a secure communication system to enhance system reliability and resilience against cyber–physical threats. To verify system performance, we first used a mug as a test object to validate the robotic arm’s posture control, motion accuracy, and trajectory consistency. The platform was then extended to quadruped robot maintenance. Experimental results show that the proposed YOLO11 model achieved an mAP@0.5 of 0.995 with reduced computation time. In addition, real experiments demonstrated that the system accurately located the target, with average planar and depth deviations of 0.0277 m and 0.0301 m, respectively. Following the physical proxy validation, the workflow was extended to a simulated quadruped-robot maintenance scenario, in which screw access reachability, trajectory feasibility, and collision-free manipulator motion were evaluated. DIATwin was used for kinematic modeling, trajectory generation, and physical robot script generation, whereas NVIDIA Omniverse was employed as a high fidelity visualization and physics-verification environment using the URDF model exported from DIATwin. The trajectory-transfer experiments demonstrated that digital-twin-generated paths could be successfully transferred to and executed by the physical robotic arm across the evaluated test configurations. Therefore, the proposed system demonstrates the feasibility of integrating vision-guided manipulation, secure communication, and digital-twin-based pre-deployment verification for future smart, automated, and cyber–physical robotics systems.

## 1. Introduction

With rapid advances in artificial intelligence (AI) and automation, robots are increasingly applied across diverse industries. In particular, quadruped robots have become a focal point of research and development due to their excellent mobility and other advantages [1,2,3,4,5]. Their versatile design enables them to perform tasks that are difficult for wheeled or tracked robots. Despite their expanding use in military, industrial, agricultural, medical, and research fields, quadruped robots still require improved autonomy, decision-making, and self-maintenance to reach their full potential [1,4,5]. Traditional maintenance often suffers from low efficiency when handling high-precision tasks and is prone to human error, increasing costs and affecting product quality [6,7,8]. Hence, to overcome these challenges, the industry must adopt automated maintenance technologies to enhance operational safety, precision, and efficiency. Thus, to realize maintenance operations, this study constructs two virtual simulation scenarios: one performs localization and arm path planning for a mug and is subsequently used for physical verification; the other simulates localization and operational actions for the external housings of a quadruped robot’s limbs. The vision-guided workflow and manipulator motion paths established in the mug scenario can be extended to quadruped-robot maintenance tasks as a technical basis for validating simulated task paths. The validation was intentionally designed as a two-stage process. In the first stage, a ceramic mug was selected as a simplified proxy target because its regular geometry, visually distinctive features, and clearly defined handle provide a controlled reference for evaluating the complete perception-to-manipulation pipeline, including object detection, RGB-D localization, camera-to-robot coordinate transformation, inverse kinematics, and robotic-arm motion execution. This simplified target reduces geometric complexity and collision risk during initial validation while retaining the fundamental requirements of target recognition, three-dimensional localization, and end-effector approach planning. After establishing the basic perception, localization, coordinate-transformation, and motion-transfer functions using the proxy object, the same workflow was extended to a more geometrically constrained quadruped-robot maintenance scenario in simulation. The mug was therefore not intended to reproduce the mechanical interaction involved in screw removal, but rather to provide a controlled intermediate validation target for the perception-to-motion pipeline before evaluating maintenance-oriented reachability and trajectory feasibility. Accordingly, the present physical experiments validate the fundamental vision-guided localization and virtual-to-real motion-transfer framework, whereas screw-removal operations on the quadruped robot are evaluated as simulation-based pre-deployment tasks.

Moreover, for physical validation, we use the YOLO (You Only Look Once) object detection model. Several versions of YOLO have been studied in previous work for object detection [9,10,11]. In this study, YOLOv11 is employed for its faster inference speed, lower parameter count, and improved accuracy, making it well-suited for real-time applications [12]. Besides, depth information is obtained via a depth camera, and target localization is completed through camera-manipulator coordinate transformation. This approach demonstrates the feasibility of integrating virtual simulation with physical verification and provides a novel maintenance method for future intelligent-maintenance scenarios. However, real-world experiments revealed noticeable planar and depth deviations caused by cumulative errors in sensor calibration and arm control. Moreover, to overcome these problems, the proposed system incorporates a digital twin simulation, which models the robotic arm and the maintenance environment virtually. This enables pre-validation of path planning, posture control, and workflow feasibility, reducing risk and improving precision before physical execution. In the initial validation phase, a mug was used as a simplified target to test robotic arm positioning and motion control. The arm’s movement from its initial position to designated target points was simulated, and the resulting trajectories were applied to the physical arm. Furthermore, the system was extended to quadruped robot maintenance, simulating screw locations and disassembly, demonstrating the flexibility of the digital twin for complex multi-step operations.

**Figure 1:**
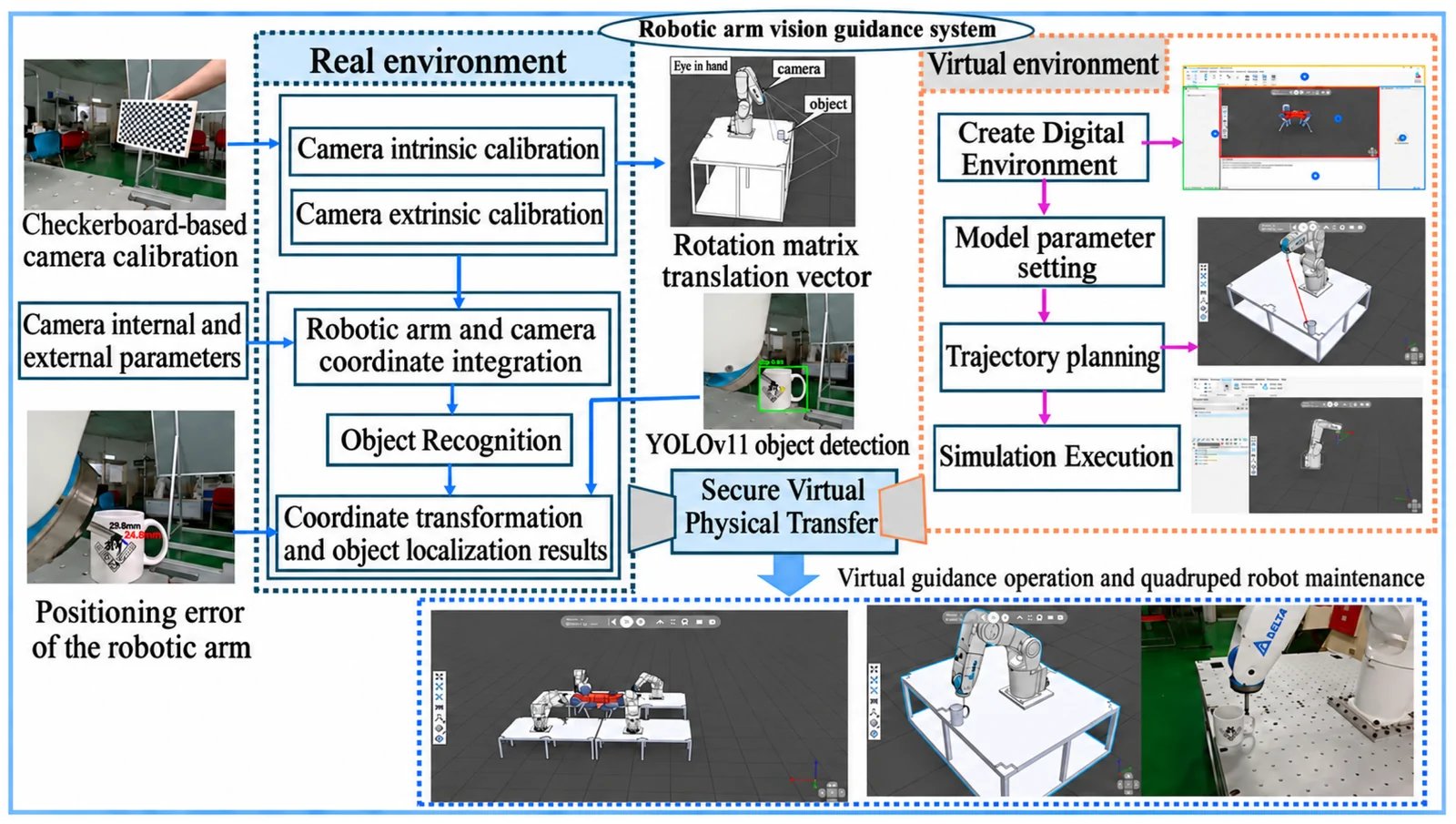
Overall system architecture and workflow of the proposed system.

Figure 1 shows the proposed system’s architecture with real and virtual environments for integrated validation. In the real environment, a checkerboard-based method is used for camera calibration, obtaining intrinsic parameters, and the relative pose between the camera and robotic arm. Based on the calibrated parameters, camera–robot coordinate integration is established to ensure consistent spatial mapping. YOLO11 then detects maintenance targets, and the detected image coordinates are transformed into the robotic arm coordinate system to achieve precise object localization. The resulting positioning errors are monitored to evaluate system accuracy. In the virtual environment, a corresponding digital twin of the robotic arm and maintenance workspace is constructed. Model parameters are configured to match the physical system, and trajectory planning and motion simulation are performed. The planned trajectories are executed within the simulation to verify motion safety and stability before deployment. Through a virtual–physical transfer mechanism, validated trajectories from the digital twin are transferred to the real robotic arm, enabling pre-checked maintenance operations and reducing trial-and-error. Therefore, the proposed framework provides a platform for evaluating vision-guided manipulation and digital-twin-based pre-deployment verification for robotic maintenance applications.

In this paper, a vision-guided robotic-arm and digital-twin framework is developed as a pre-validation platform for future automated quadruped-robot maintenance. The proposed system integrates YOLOv11 with robotic manipulation and secure digital twin simulation, enabling autonomous target localization, precise path planning, and pre-validation of motion trajectories. System performance was first validated using a mug, then extended to robot maintenance. Experimental results confirm that the proposed YOLOv11 method improves detection accuracy and reduces computation time, while real-environment tests demonstrate reliable target identification and positioning. Hence, the proposed framework provides a basis for the development of safe and efficient quadruped-robot maintenance systems, while physical validation of fine screw-removal operations remains necessary before full autonomous deployment. The main contribution of this work:

- We develop a vision-guided robotic-arm framework integrating YOLO11-based perception, RGB-D localization, robotic manipulation, and digital-twin-based trajectory pre-validation for future quadruped-robot maintenance applications.
- We develop a 3D target-localization pipeline combining YOLO11 detection with RGB-D depth sensing and camera-to-robot coordinate transformation, and experimentally characterize its positioning accuracy and systematic localization offset.
- We implement and experimentally evaluate a secure communication layer using TLS-protected MQTT and certificate-based authentication. The Raspberry Pi 4 gateway test demonstrated successful encrypted communication, supporting the feasibility of the proposed approach for high-level robotic command and telemetry exchange.
- We establish a simulation-based quadruped-maintenance workflow for evaluating manipulator reachability, end-effector approach orientation, trajectory feasibility, and collision-free task execution prior to physical deployment.

The remainder of this study is organized as follows. Section 2 presents the experimental setup, Section 3 describes the proposed methodologies, Section 4 presents the results and discussion, and Section 5 concludes the study.

## 2. Experimental Setup

**Figure 2:**
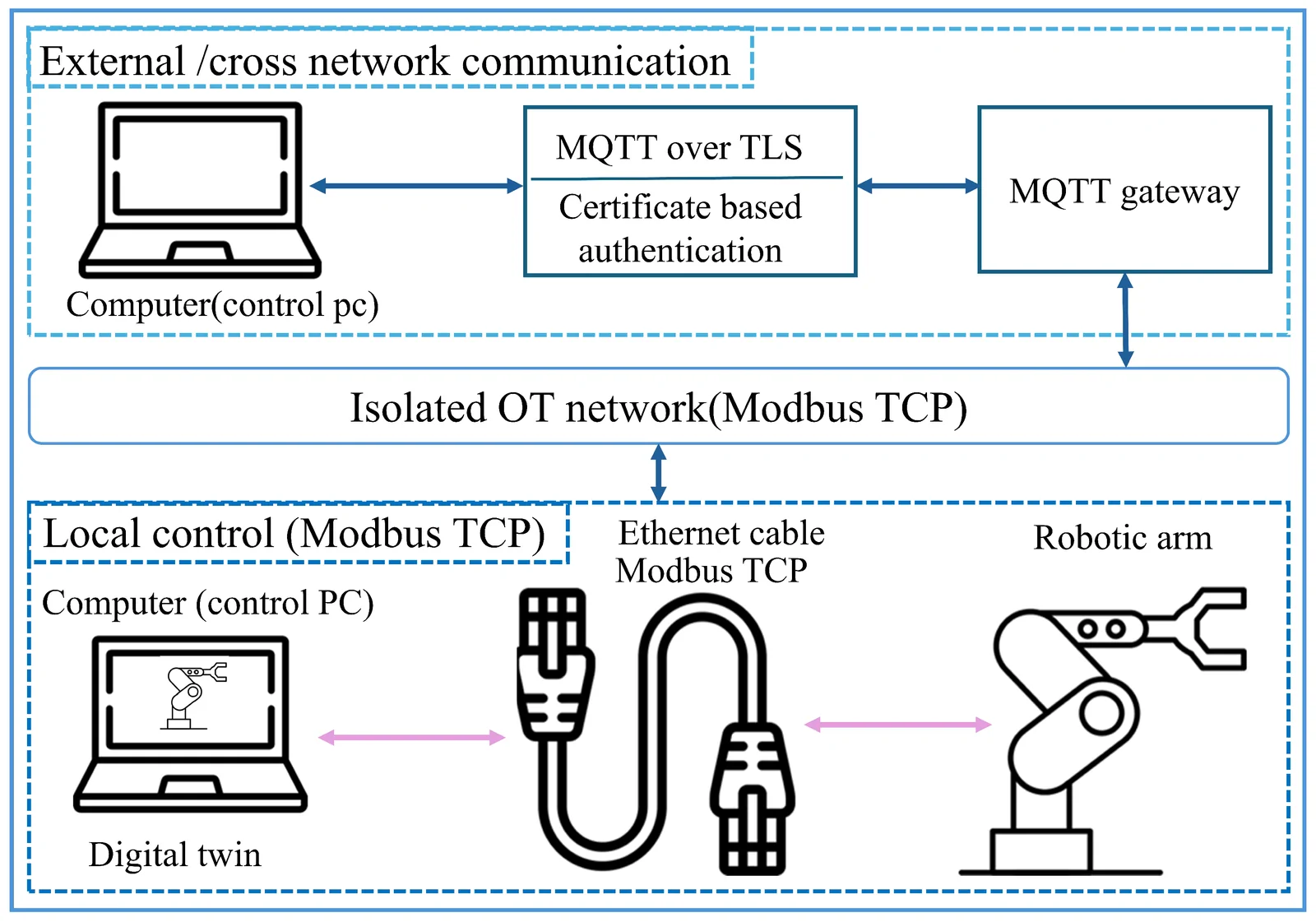
Secure robotic arm communication and control architecture.

The experimental setup of the proposed system integrates hardware, software, and simulation components for object detection, manipulation, and validation tasks. The hardware system comprises an Intel RealSense D455 depth camera (Intel Corporation, Santa Clara, CA, USA) for all RGB-D data capture and 3-D coordinate extraction, and a DRV70L7A61 six-axis industrial robotic arm (Delta Electronics, Inc., Taipei, Taiwan) responsible for executing pick-and-place and maintenance motion primitives. Communication between the robotic arm and the control PC (personal computer) was established via Ethernet-based Modbus TCP for command and telemetry exchange, with TCP sockets managing high-level task control and motion scripts uploaded to the robot controller when required. A DIATwin-based digital twin replicates the workspace, enabling trajectory planning and validation before real-world execution. Moreover, YOLOv11 is used for object detection. Figure 2 shows the secure communication and control architecture of the six-axis robotic arm with its digital twin. The digital twin on the control PC communicates with the robotic arm controller via Modbus TCP over Ethernet, with the PC as client sending commands and the controller as server providing real-time feedback. In the present implementation, the Modbus TCP link was confined to the local OT network. Communication requiring external or cross-network access was instead routed through an MQTT gateway, where TLS encryption and certificate-based authentication were applied. In the secure communication testbed, a Raspberry Pi 4 (Raspberry Pi Ltd., Cambridge, UK) was configured as the MQTT intermediary to evaluate the feasibility and communication overhead of authenticated and encrypted data transfer. Packet-level traffic was captured to verify successful TLS session establishment and encrypted application-data exchange.

## 3. Proposed Methodologies

### 3.1. Data Collection and Annotations

The data used in this study was collected using an Intel RealSense D455 RGB-D camera (Intel Corporation, Santa Clara, CA, USA) mounted on the robotic arm. The primary target object was a ceramic mug, selected for its simple yet distinct geometry, which enables testing of detection and depth estimation performance. To ensure comprehensive coverage, RGB and depth frames were captured from multiple viewpoints and arm configurations, with deliberate variations in distance, orientation, and illumination. These variations simulate realistic operating environments and enable the model to generalize effectively to different viewing conditions. Each image was synchronized with its corresponding depth frame and calibrated using the D455’s intrinsic parameters to ensure geometric consistency. The dataset contained one target class, namely the ceramic mug and the background. We collected 125 original RGB images under different viewpoints, object orientations, camera-to-object distances, and illumination conditions. The original dataset was divided into 87 training images, 19 validation images, and 19 test images. The training subset was subsequently augmented using rotation, translation, and brightness adjustment, increasing the training set to a total of 212 images. The validation and test subsets remained unchanged and contained only original, non-augmented images. Therefore, the final dataset contained 250 images, comprising 212 training images, 19 validation images, and 19 test images.

Moreover, data preprocessing is a crucial step in preparing data for the model to accurately recognize the target object. The quality and consistency of input images directly affect the model’s ability to learn meaningful visual features. However, real-world images often exhibit variations in angle, lighting, and noise, which can degrade model performance if not properly addressed. Furthermore, to address these issues, the image preprocessing pipeline comprised two main stages: image annotation and data augmentation. Annotation: ensures a clear definition of target regions for supervised learning. Data augmentation increases dataset diversity through transformations like rotation and brightness changes, improving robustness to varying and unseen conditions.

### 3.2. Data Annotation and Augmentation Method

**Figure 3:**
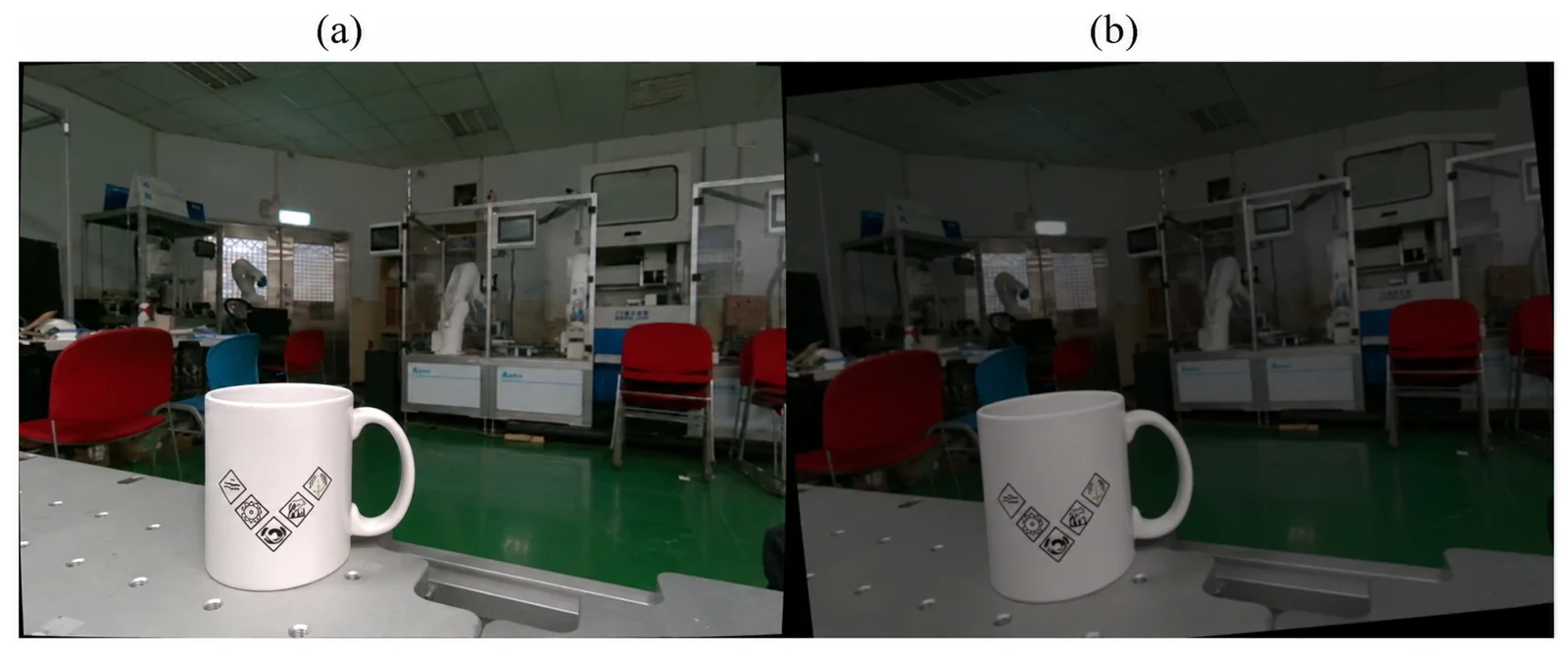
Sample images: (**a**) original image; (**b**) augmented image.

In this study, LabelImg was used to annotate the data by efficiently defining object regions with rectangular bounding boxes. Each annotated image generates a corresponding YOLO-format text file, where each line represents an object instance and includes key parameters. All coordinate values are normalized within the range of 0 to 1, taking the top-left corner of the image as the origin. This normalization allows consistent training across images with varying resolutions. Although LabelImg supports only rectangular bounding boxes and requires manual labeling, it was chosen for this study due to its simplicity, flexibility, and full compatibility with YOLO-based deep learning frameworks, making it effective and reliable for preparing high-quality training data. Additionally, data augmentation enhances the model’s recognition of the mug, increasing training sample diversity and helping it adapt to different visual and environmental conditions. The augmentation operations included rotation to simulate different viewing angles, translation to account for positional shifts, and brightness adjustment to handle varying illumination. Data augmentation was applied exclusively to the training subset after the original dataset had been partitioned. The original training images were augmented to produce a final training set of 212 images. The 19 validation images and 19 test images remained unchanged and contained only original, non-augmented images. Figure 3a shows examples of the original images, whereas Figure 3b shows corresponding augmented samples.

### 3.3. Proposed YOLO11 Detection Model

In the fast-evolving fields of AI and vision technology, object detection has emerged as a crucial task with broad applications [9,13]. However, in real-time applications, traditional object detection methods are computationally intensive [12,14]. On the other hand, the YOLO family delivers real-time performance while maintaining high accuracy. However, earlier versions of YOLO still had limitations, such as slower speeds and less efficiency [15]. Moreover, YOLO11 addresses these challenges by offering faster performance and higher accuracy, making it a more effective solution for modern object detection tasks. The YOLO11 architecture incorporates key architectural enhancements, including an enhanced feature fusion (Neck) mechanism and improved spatial attention mechanisms. In addition, YOLO11 is an advanced network model that brings significant improvements over its predecessors [15,16]. The C3K2 module (cross stage partial with kernel size 2) enhances the extraction of complex features. In addition, the SPPF module (Spatial Pyramid Pooling–Fast), an optimized version of the SPP module, captures object features at multiple scales. The C2PSA module (convolutional block with parallel spatial attention) further integrates channel and spatial information and incorporates a multi-head attention mechanism, thereby strengthening the model’s ability to perceive objects. In this study, the YOLO11 nano variant was adopted as the primary detection model. The model was initialized using the pretrained yolo11n.pt weights and subsequently fine-tuned on the custom ceramic-mug dataset. The proposed model was trained using consistent training parameters, including 50 learning iterations, a batch size of 10, an input image size of 640 × 640 pixels, and an initial learning rate of 0.01. After training, the model was capable of real-time inference, generating predicted bounding boxes, target classes, and corresponding confidence scores. The reported detection performance was evaluated using precision, recall, F1-score, mAP@0.5, and mAP@0.5:0.95.

**Figure 4:**
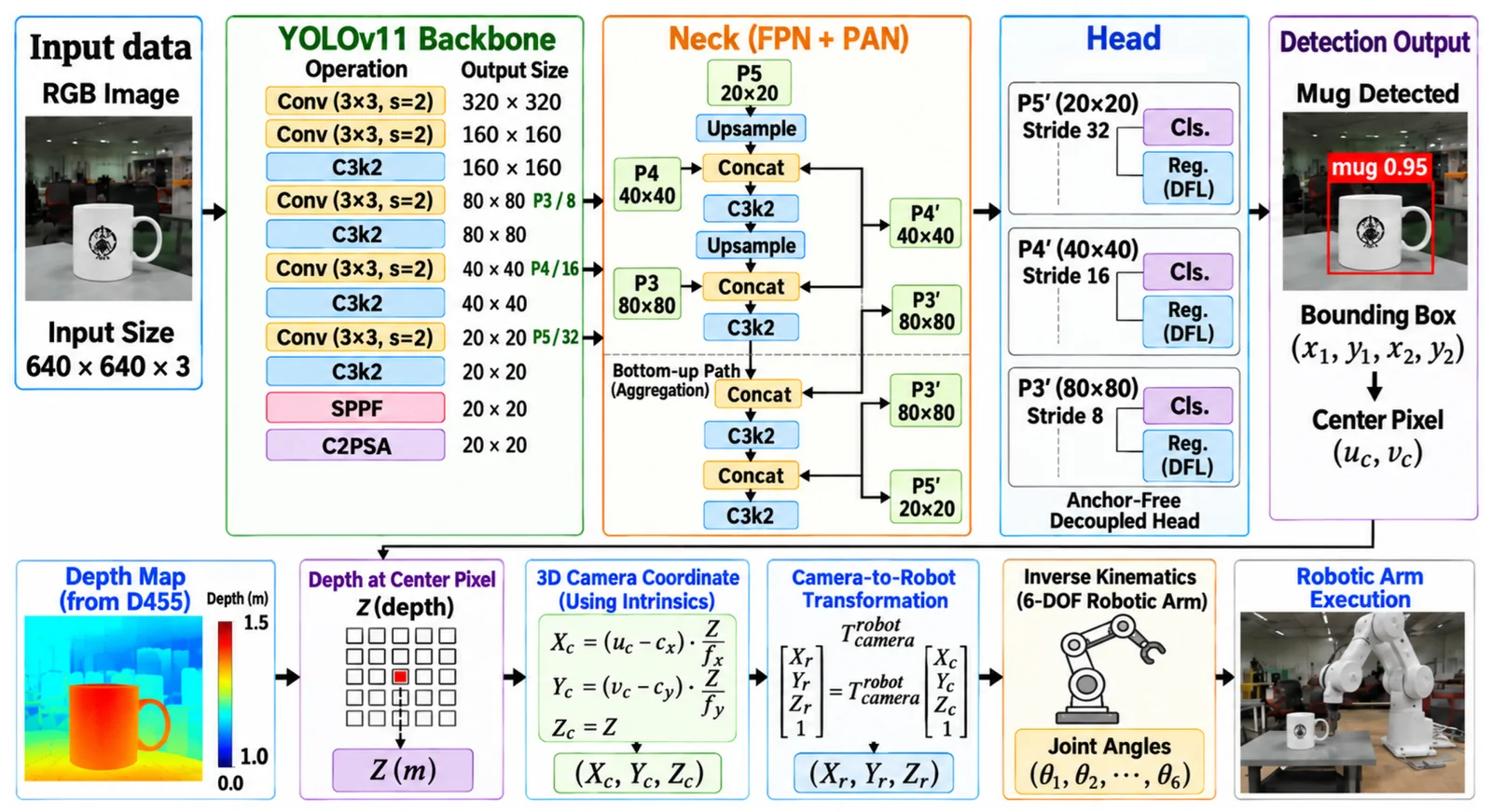
YOLO11-based vision-guided 3D target localization and robotic manipulation framework.

Moreover, Figure 4 shows the YOLO11-based vision-guided 3D target localization and robotic manipulation framework. RGB images acquired by the Intel RealSense D455 are processed by the YOLO11 detector, whose backbone employs C3k2, SPPF, and C2PSA modules for hierarchical and multi-scale feature extraction. The FPN–PAN neck aggregates features from the P3, P4, and P5 levels, while the anchor-free decoupled detection head predicts the target class and bounding-box coordinates at multiple spatial resolutions. The center pixel of the detected target is subsequently associated with the corresponding D455 depth measurement and converted into a three-dimensional camera-frame coordinate using the calibrated intrinsic parameters. The resulting coordinate is transformed into the robotic-arm reference frame using the camera-to-robot transformation matrix, after which inverse kinematics determines the six joint angles required for target localization and robotic-arm execution. Accordingly, the analytical workflow can be expressed as a sequential mapping from image-space detection to camera-frame three-dimensional coordinates, followed by rigid transformation into the robot base frame and inverse-kinematic mapping to the manipulator joint space. This formulation provides the mathematical connection between visual perception and robotic execution and forms the basis for transferring the same target and trajectory representation into the digital-twin environment.

### 3.4. Proposed Digital Twin Method

**Figure 5:**
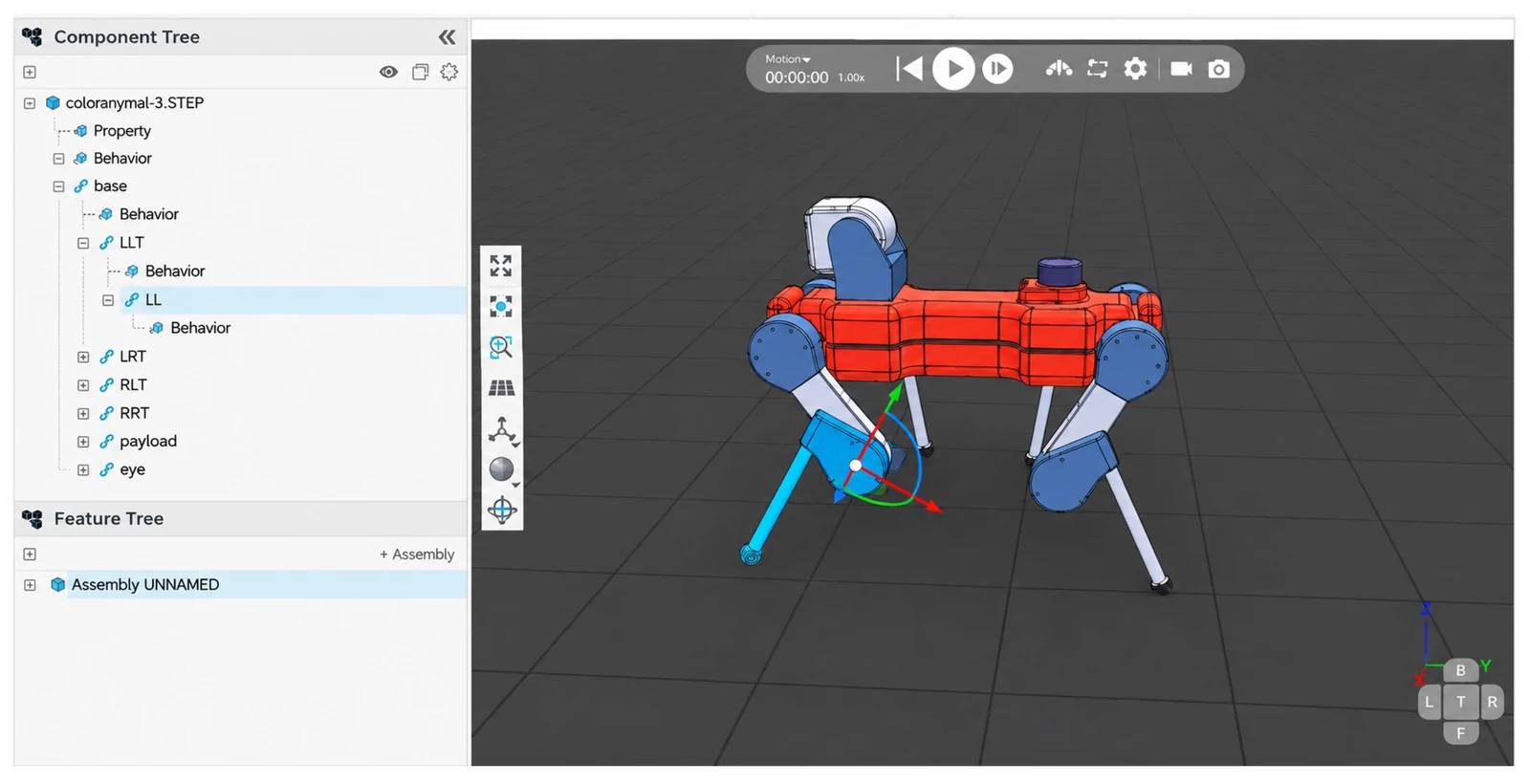
Virtual quadruped robot construction.

In smart manufacturing and autonomous robotics, digital twins have become essential for improving predictability, visualization, and real-time adaptability [17,18,19,20]. Digital twins are widely adopted across manufacturing, robotics, healthcare, and others [21,22,23,24]. Moreover, a digital twin creates a virtual replica of a physical system, enabling real-time mapping of its states and behaviors [25,26,27,28]. To enable integrated cyber–physical control, a digital twin environment was created that incorporates the robotic arm’s structure, control logic, and workflow for synchronized motion and object interaction. The digital twin platform used is DIATwin by Delta Electronics. Figure 5 shows the virtual construction of the quadruped robot. The development began by importing the CAD components and converting them into a kinematic linkage representation. Subsequently, the primary body link and all limb components were established. After configuring each link and joint, a controller module was created, and the linkage components were integrated into the control framework to enable motion execution in the virtual environment. The robotic arm has a hierarchical structure, with each joint defined relative to the previous one, matching the real arm while enabling flexible control. The two digital-twin platforms used in this study have complementary rather than redundant roles. DIATwin serves as the primary kinematic and robot-control-oriented digital twin. It is used to construct the manipulator and quadruped linkage models, configure joint relationships and coordinate frames, define target points, generate point-to-point trajectories, and export Lua motion scripts for execution by the physical Delta robotic arm. NVIDIA Omniverse serves as a secondary high-fidelity visualization and physics-verification environment. The URDF model generated from DIATwin is imported into Omniverse to preserve the robot kinematics, joint constraints, coordinate frames, and collision geometry while enabling RTX-based rendering, workspace visualization, reachability inspection, and collision verification. In the current workflow, physical robot commands are generated through the DIATwin/control-PC workflow; Omniverse is used for offline pre-deployment verification rather than direct closed-loop control of the physical manipulator. The digital-twin model includes the manipulator linkage hierarchy, coordinate frames, link-mass parameters, joint rotation ranges, base/end-effector definitions, and collision bounding geometry. The physical DRV70L7A61 joint limits were used as reference parameters. Therefore, the digital twin is used primarily for kinematic, workspace, reachability, and collision-feasibility analysis rather than as a fully validated dynamic replica.

**Figure 6:**
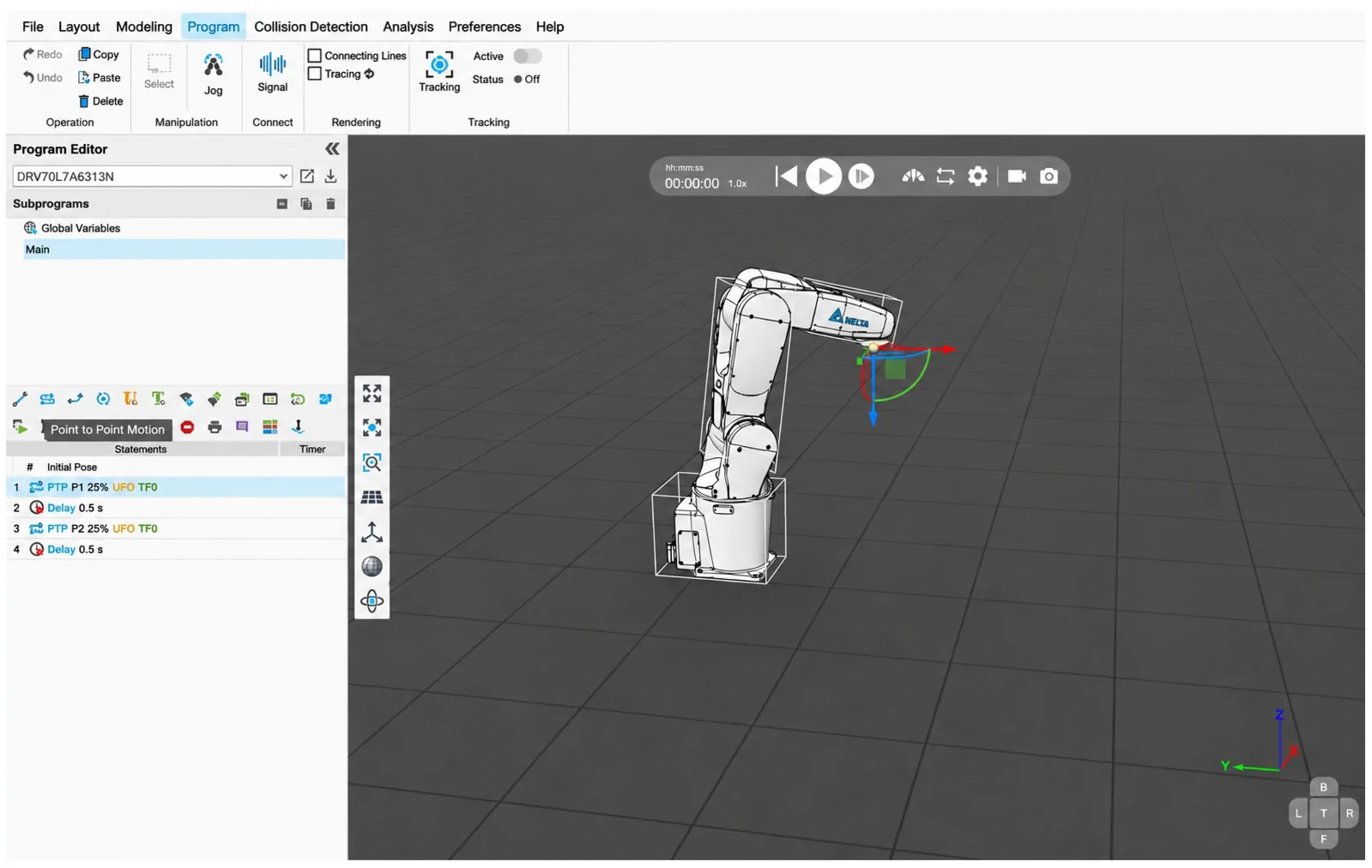
Virtual robotic arm and path arrangement.

Linkage components were integrated to define the base and end-effector references for path planning. Target points were adjusted by joint angles, and the point-to-point motion function generated the corresponding movements, as shown in Figure 6. The transformation matrices in Equations (1) and (2) represent the first through sixth joints, and yield the end-effector pose Equation (7).

(1) T10=cosθ1−sinθ10a1cosθ1sinθ1cosθ10a1sinθ1001d10001

(2) T21=cosθ20−sinθ2a2cosθ2sinθ20cosθ2a2sinθ20−10d20001

(3) T32=cosθ3−sinθ30a3cosθ3sinθ3cosθ30a3sinθ3001d30001

(4) T43=cosθ40−sinθ4a4cosθ4sinθ40cosθ4a4sinθ40−10d40001

(5) T54=cosθ50sinθ5a5cosθ5sinθ50−cosθ5a5sinθ5010d50001

(6) T65=cosθ60−sinθ6a6cosθ6sinθ60cosθ6a6sinθ60−10d60001

(7) T60=T10·T21·T32·T43·T54·T65

The sequence of homogeneous transformation matrices defines the forward-kinematic relationship between the robot base and end-effector frames, while inverse kinematics is used to obtain the corresponding joint configuration for a desired target pose. These relationships provide the mathematical basis for maintaining consistent pose definitions between the physical robotic arm and the digital-twin model. After constructing the mechanical model, development proceeded to the perception system. The virtual camera is configured with intrinsic parameters and a field of view to replicate the real lens’s optical behavior. Once configured, the calibrated virtual camera supports image recognition, object tracking, and spatial reconstruction, enhancing the digital-twin robot’s environmental perception. Moreover, to verify transfer to the physical arm, a target point on the mug handle was set in the virtual environment. Based on this reference, feasible motion trajectories were planned and tested at five different positions and orientations. The planned path was exported as a Lua script compatible with the Delta Electronics robot language interface, enabling users to inspect, edit, and execute the commands. Running the script on the physical arm validated the simulated trajectories, confirming that the real arm accurately reproduced the motions and demonstrating effective cyber-physical integration. The two digital-twin platforms employed in this study perform complementary functions. DIATwin is the primary robot-modeling and control-oriented platform and is used to construct the robotic-arm and quadruped linkage models, configure joint relationships and coordinate frames, define target points, generate point-to-point trajectories, and export Lua motion scripts for execution by the physical Delta robotic arm. NVIDIA Omniverse is used as a secondary high-fidelity visualization and physics-verification environment. The URDF model exported from DIATwin is imported into Omniverse while preserving kinematic relationships, joint constraints, coordinate frames, and collision geometry. Omniverse is then used for workspace visualization, reachability inspection, collision checking, and physics-based pre-deployment verification. In the present architecture, physical robot commands are generated through the DIATwin/control-PC workflow, whereas Omniverse does not directly control the physical robotic arm.

## 4. Results and Discussions

This section presents experimental results demonstrating accurate perception, stable manipulation, and seamless virtual–real integration through vision-based detection, robotic control, and digital twin simulation. The proposed vision-guided robotic arm system was developed and evaluated using a workstation equipped with an NVIDIA RTX 2070 GPU with 8 GB of VRAM and 32 GB of DDR4 RAM. The object-detection models were developed in Python 3.10 packages, using the Ultralytics YOLO framework. DIATwin was used for digital-twin modeling and trajectory planning. Standard metrics, precision, recall, F1-score, and mean average precision (mAP) were used to evaluate the proposed method, computed as follows [29,30]:

(8) $$\mathrm{Precision}=\frac{TP}{TP+FP}$$

where TP True Positive, TN True Negative, FP False Positive, and FN False Negative.

(9) $$mAP=\frac{\sum_{n=1}^{N}AP_{n}}{N}$$

N is the total classes, APn average precision for class ‘n’.

### 4.1. YOLO11 Detection Model Performance

Table 1 shows the performance of YOLOv11 and YOLOv10. Both achieve better precision and recall, but YOLOv11 attains a higher F1-score and more stable mAP, demonstrating more reliable and comprehensive detection. YOLO11n exhibited an inference time of 8.47 ms, corresponding to 118 FPS, whereas YOLOv10n exhibited an inference time of 10.52 ms, corresponding to 95.1 FPS. Therefore, under the present experimental conditions, YOLO11 achieved higher detection performance than YOLOv10 on the evaluated dataset.

**Figure 7:**
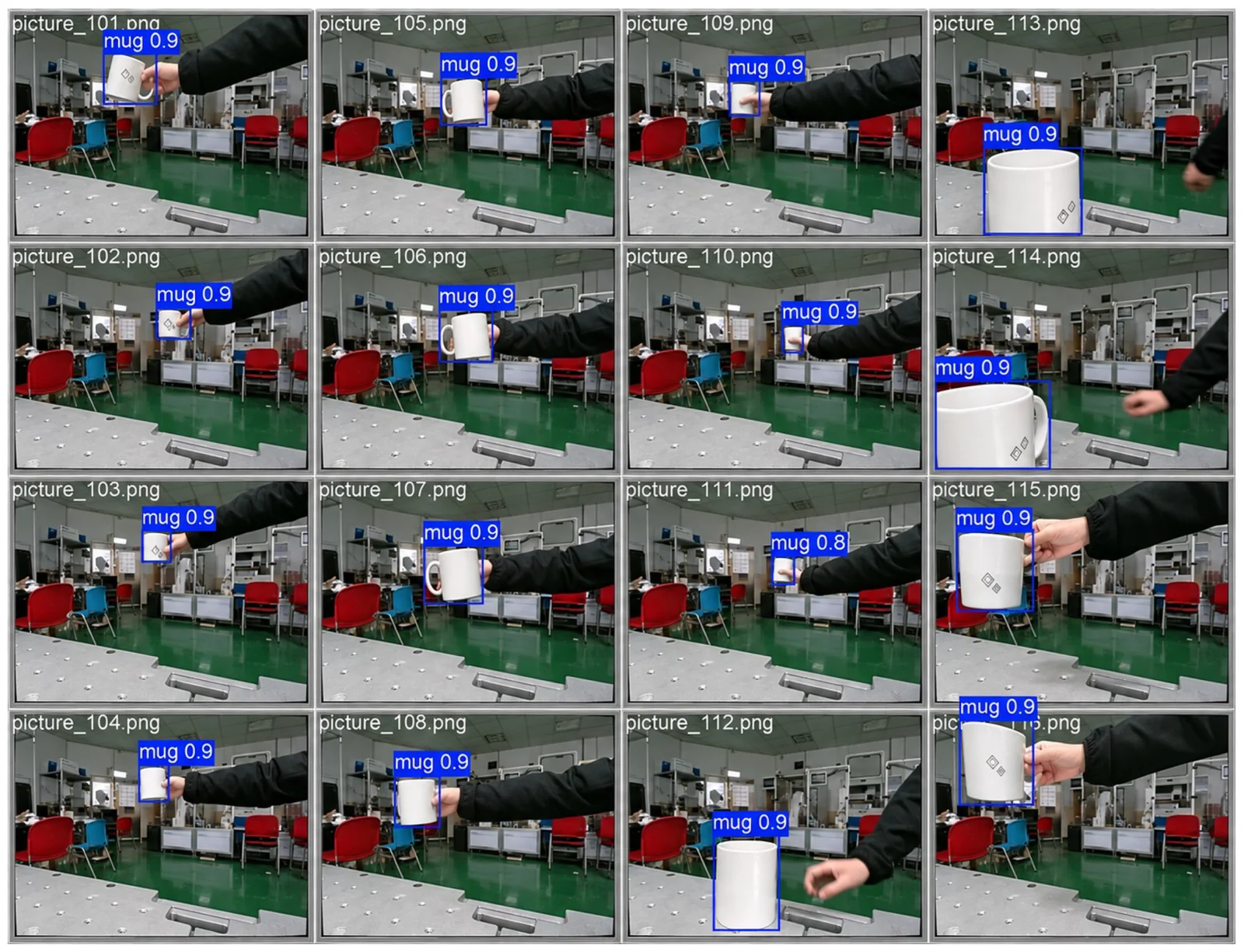
YOLOv11 mug detection results under different viewing angles.

Moreover, Figure 7 illustrates the object detection performance of the YOLOv11 model on a series of test images containing a ceramic mug under varying poses, orientations, and motion conditions. The bounding boxes and confidence scores in the images show that YOLOv11 consistently identifies the mug with high confidence values ranging from 0.8 to 0.9, demonstrating robust recognition performance under varying orientations. This indicates that the trained YOLOv11 model achieves stable detection performance, effectively recognizing the target object across different spatial perspectives. The results indicate that YOLOv11 maintained stable mug detection across the tested variations in object position, orientation, viewpoint, and background conditions. The model maintains precise bounding-box localization despite changes in hand movement, object displacement, and dynamic backgrounds, highlighting its stability and reliability for downstream robotic perception and manipulation tasks.

**Figure 8:**
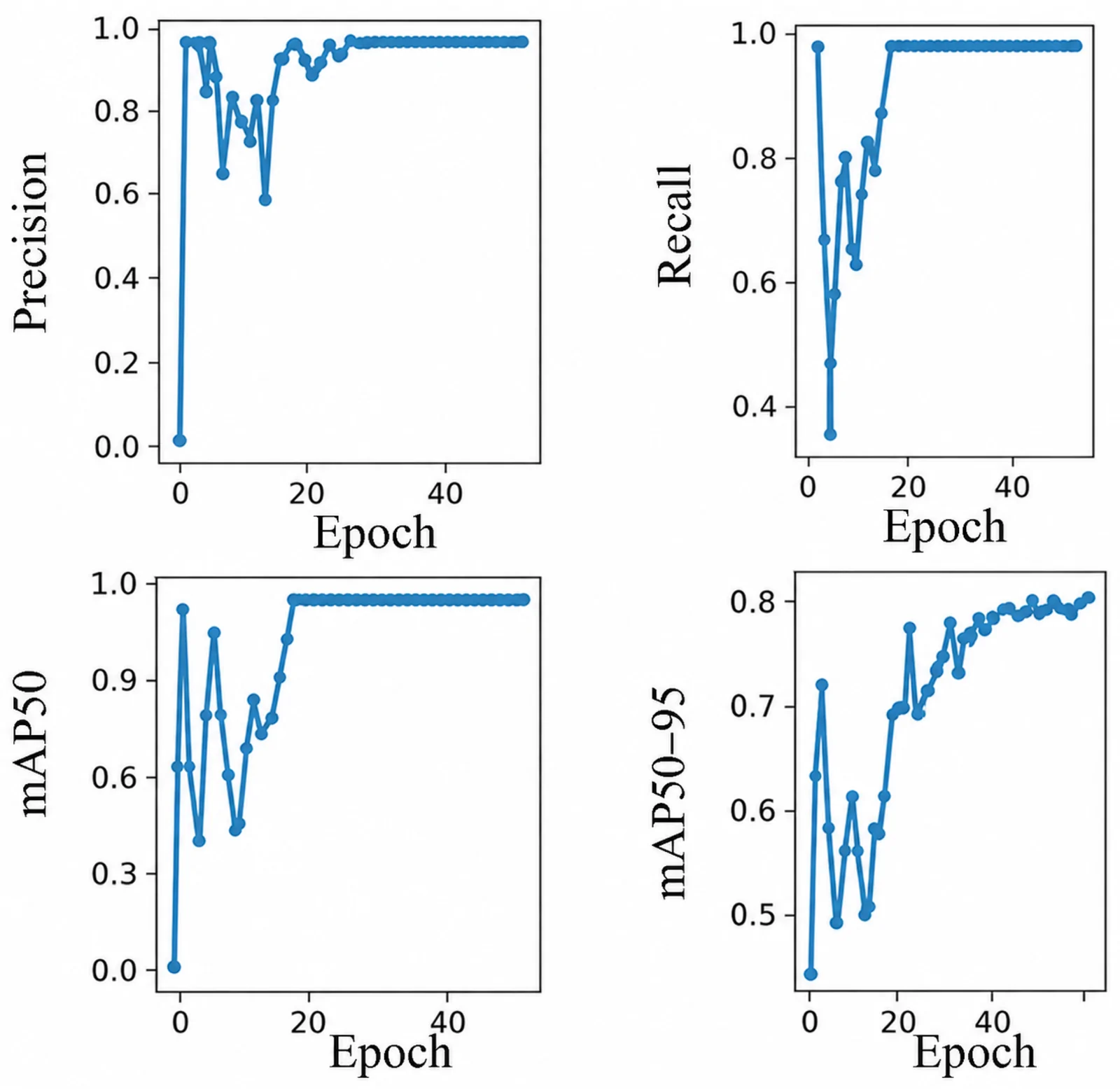
Proposed model performance in terms of precision, recall, and mAP.

Figure 8 shows the model’s performance in precision, mAP, and recall, demonstrating stable and consistent improvement across all metrics. In the early stage, precision, recall, and mAP fluctuate as the model adjusts, then quickly converge and stabilize, indicating consistent learning. Both precision and recall approach optimal performance, showing accurate target detection, while mAP50 rises rapidly and stabilizes, confirming robust localization. Moreover, the more stringent mAP50–95 metric improves steadily, indicating reliable detection across IoU thresholds. Hence, the results confirm that the proposed YOLOv11 achieves fast convergence, high accuracy, and stable performance under the evaluated conditions.

**Figure 9:**
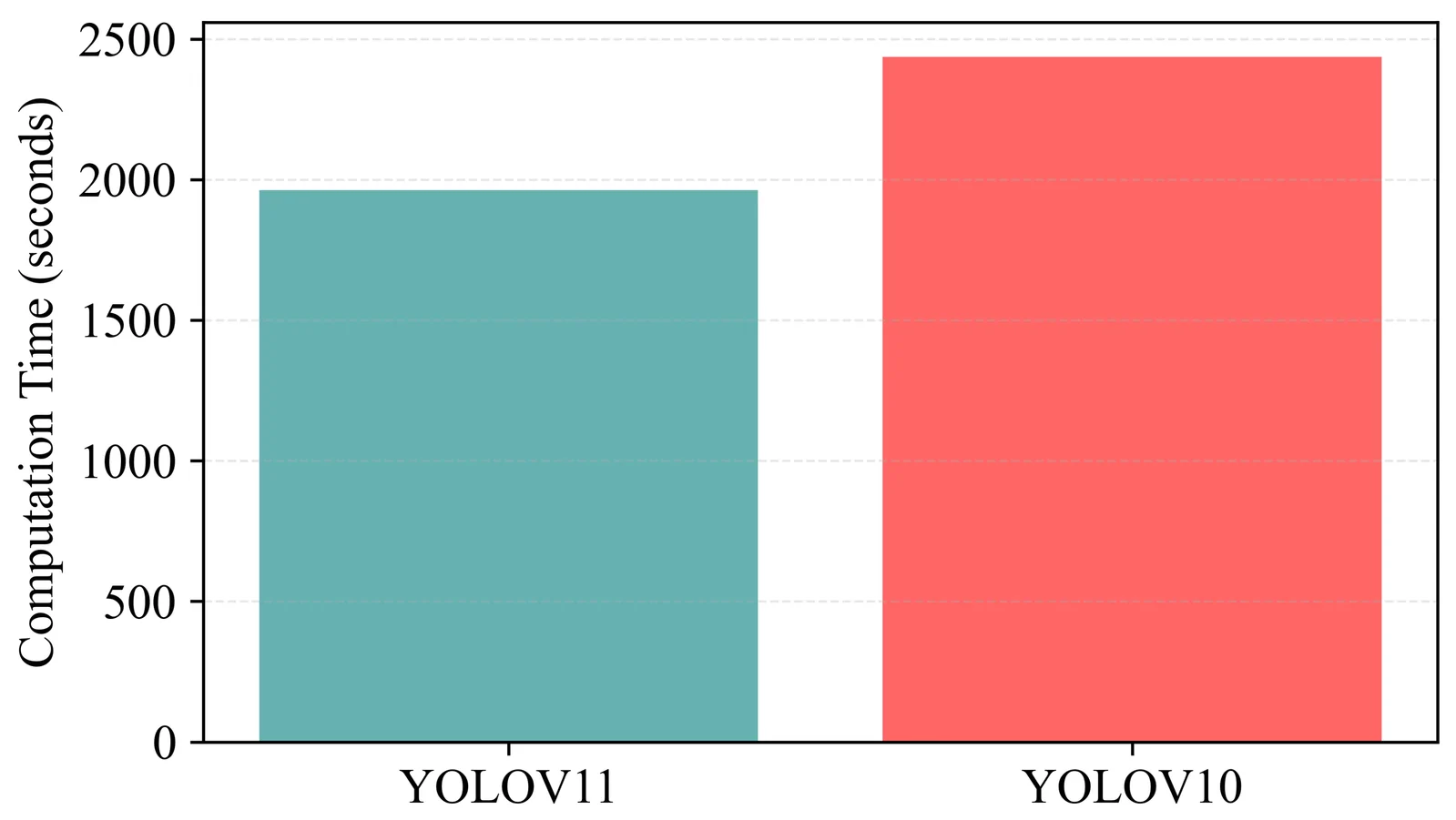
Comparison of total training time for YOLOv10 and YOLO11v.

Figure 9 compares the total training time of YOLOV10 and YOLOV11 over 50 epochs under the same experimental configuration. YOLO11 required 1961.73 s for training, whereas YOLO10 required 2436.87 s. This corresponds to an approximately 19.5% reduction in total training time for YOLO11. The reported training time represents the offline model-training process and should not be interpreted as the runtime inference time. Runtime detection performance is instead evaluated using the inference time and FPS reported in Table 1.

### 4.2. YOLO11-Driven Object Localization with Robotic Arm Integration

**Figure 10:**
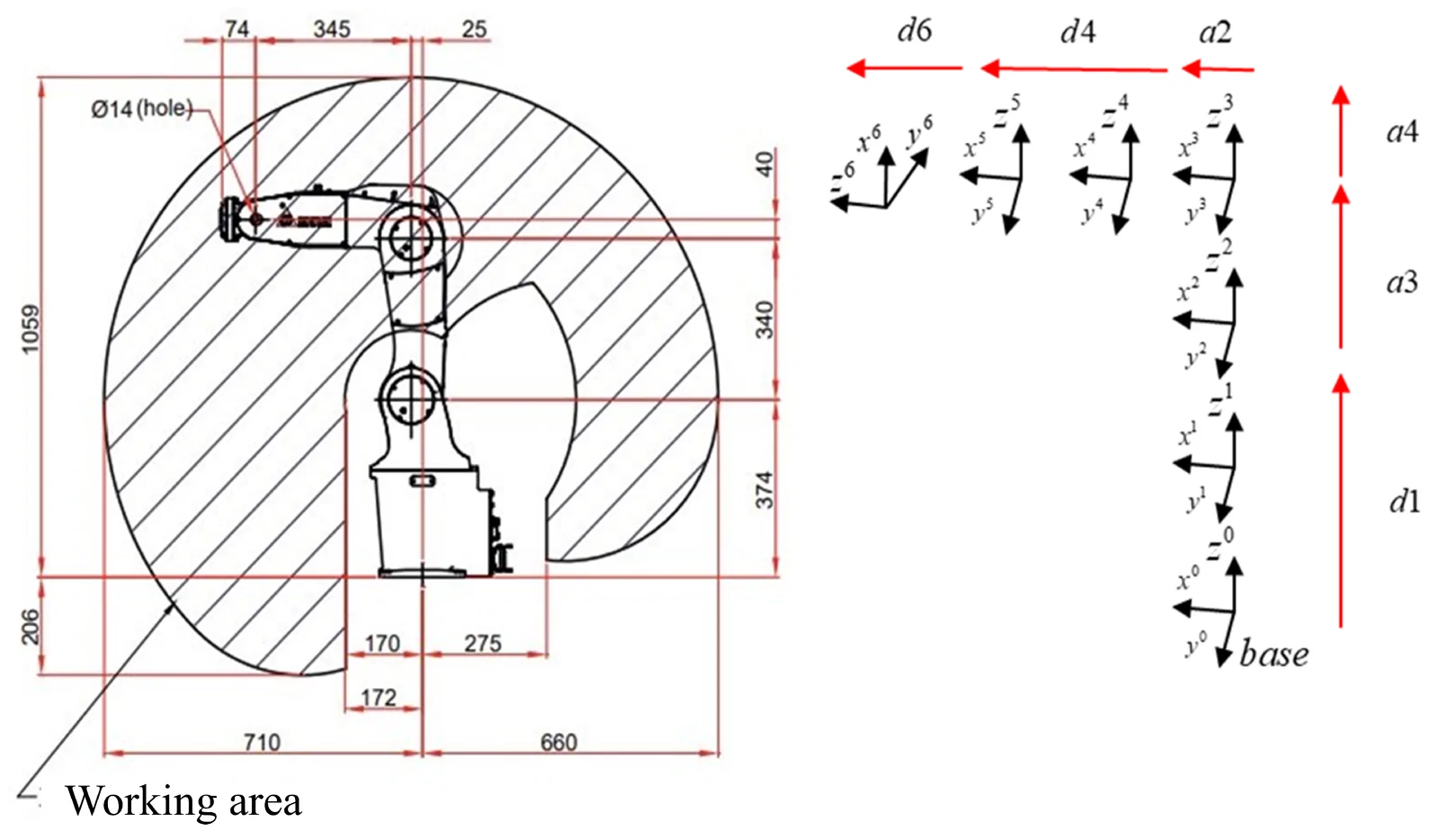
Kinematic configuration of the DRV70L7A61 six-axis robotic arm, showing the joint axes, coordinate frames, and geometric parameters used in the inverse-kinematics formulation.

Camera intrinsic calibration was performed using 15 checkerboard images acquired from different viewing angles. An Eye-to-Hand configuration was adopted, with the Intel RealSense D455 positioned within the robotic-arm workspace. Multiple checkerboard observations acquired at different robotic-arm poses, together with the corresponding end-effector poses, were used to estimate the rigid camera-to-robot transformation, including the rotation matrix and translation vector. The detected target pixel was combined with the corresponding depth measurement to obtain a three-dimensional coordinate in the camera frame, which was then transformed into the robotic-arm coordinate frame. To verify positioning accuracy, 16 tests were conducted at different targets. The model detected each object and extracted its coordinates, which were transformed into the robot’s frame using calibration parameters. The target positions were input to the inverse kinematics (IK) model to compute joint angles for execution by the robotic arm. IK determines the joint angles required for the robot to reach a desired end-effector pose, as given in Equation (10). Joint angle calculation starts with the first joint, which rotates about Z to position the manipulator in the XY plane. Given the end-effector position in Equation (10), its projection to the XY plane is Equation (11). To clarify the geometric parameters and joint-axis definitions used in the inverse-kinematics formulation, Figure 10 illustrates the kinematic configuration of the DRV70L7A61 six-axis robotic arm (a DRV70L7A61 six-axis industrial robotic arm (Delta Electronics, Inc., Taipei, Taiwan)). The coordinate frames and joint axes follow the D–H convention adopted in this study and provide the geometric basis for the joint-angle calculations in Equations (10)–(17).

(10) $$p={\left[\begin{array}{ccc} P_{x} & P_{y} & P_{z} \end{array}\right]}^{T}$$

(11) $$\theta_{1}=\arctan2\left(P_{y},P_{x}\right)$$

$\theta_{1}$ angle between the first joint axis and end-effector vector XY-plane.

(12) $$\theta_{2}=\arctan2\left(s,r\right)-\cos^{-1}\left(\frac{a_{3}^{2}+L^{2}-a_{4}^{2}}{2a_{3}L}\right)$$

where *r* XY-plane distance, *s* height, *L* joint-to-end distance.

(13) $$\theta_{3}=\cos^{-1}\left(\frac{a_{3}^{2}+a_{4}^{2}-L^{2}}{2a_{3}a_{4}}\right)$$

The end-effector rotation $R_{6}^{0}$ and the first three joints’ rotation $R_{3}^{0}$ give the third-to-end rotation $R_{6}^{3}$:

(14) $$R_{3}^{6}={\left(R_{3}^{0}\right)}^{T}R_{6}^{0}$$

(15) $$\theta_{4}=\arctan2\left(-R_{3(2,3)}^{6},\;R_{3(3,3)}^{6}\right)$$

(16) $$\theta_{5}=\arcsin\left(R_{3(1,3)}^{6}\right)$$

(17) $$\theta_{6}=\arctan2\left(-R_{3(1,2)}^{6},\;R_{3(1,1)}^{6}\right)$$

**Figure 11:**
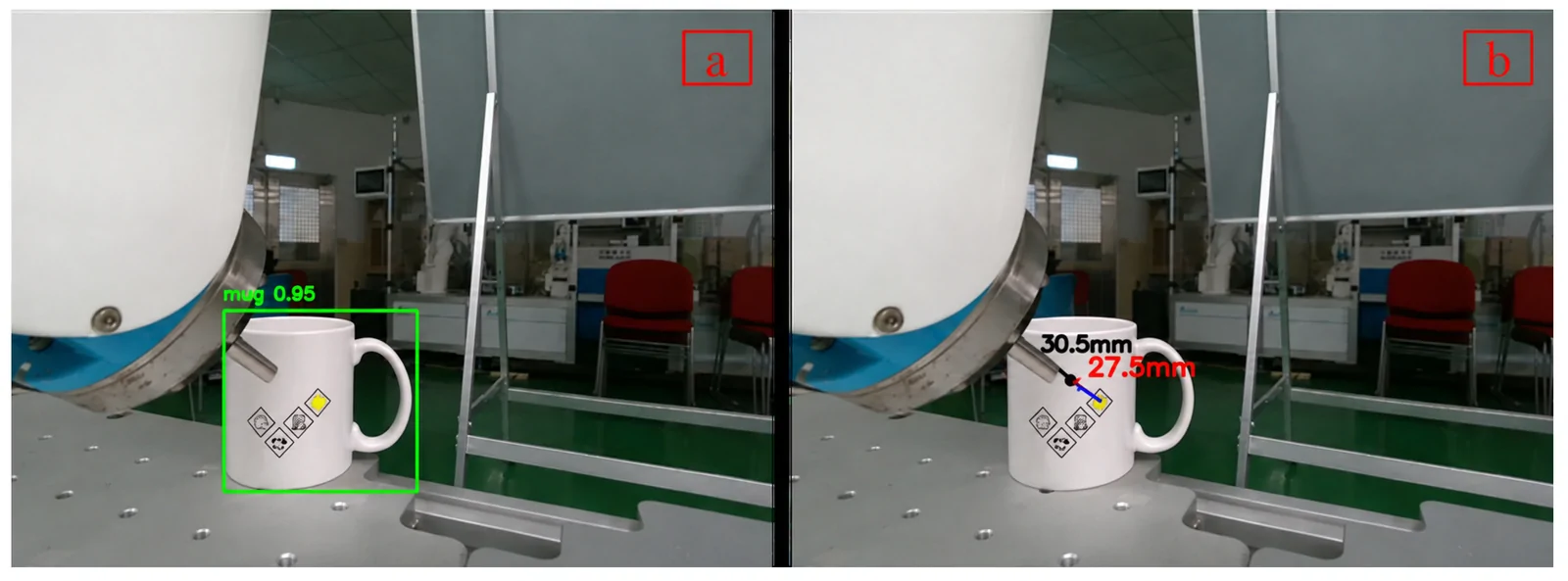
First experiment: (**a**) YOLOv11 detection, (**b**) robotic arm end-effector localization error.

Figure 11 shows the first experiment: (a) YOLOv11 detection with a confidence of 0.95, and (b) the robotic arm’s end-effector position error.

**Figure 12:**
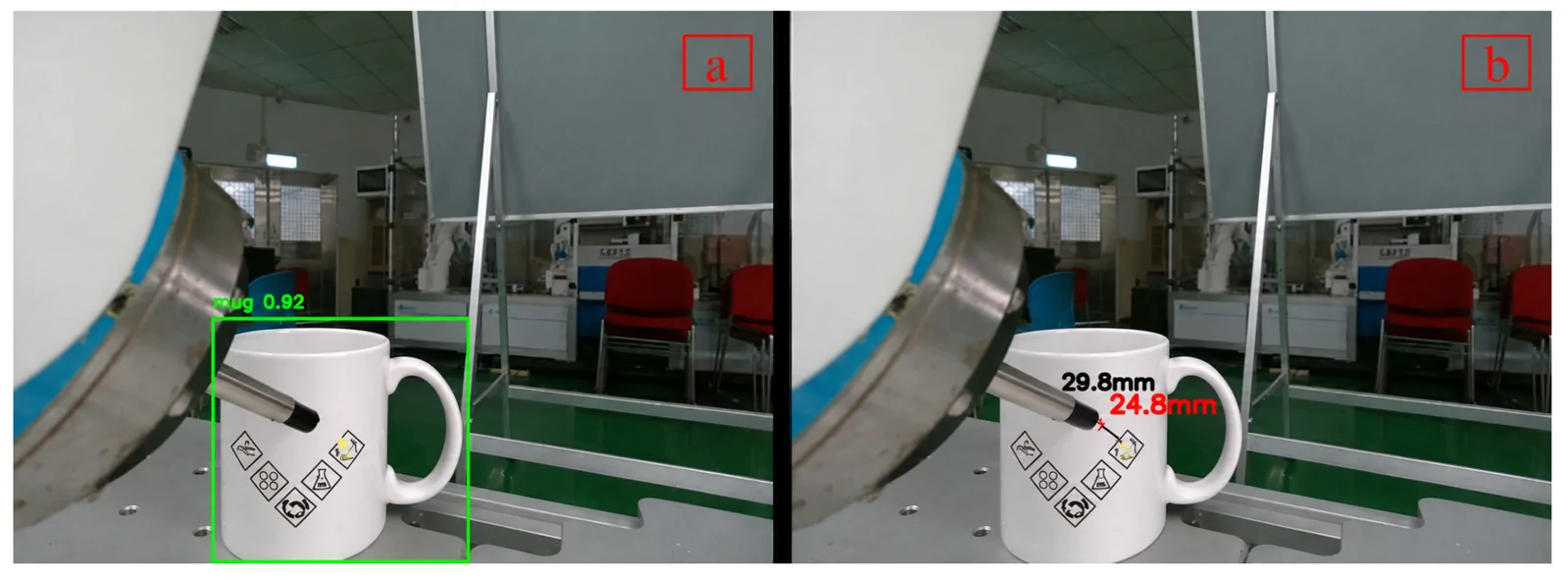
Fourth experiment: (**a**) YOLOv11 detection, (**b**) localization error.

Figure 12 shows the fourth experiment: (a) YOLOv11 detection with 0.92 confidence, and (b) the robotic arm’s end-effector localization error. The system achieved planar and depth errors of 24.8 mm and 29.8 mm, respectively, representing the lowest error among the evaluated trials. Although this result indicates repeatable coarse localization, the centimeter-scale absolute error remains insufficient for direct screw engagement and requires further fine-positioning compensation.

**Figure 13:**
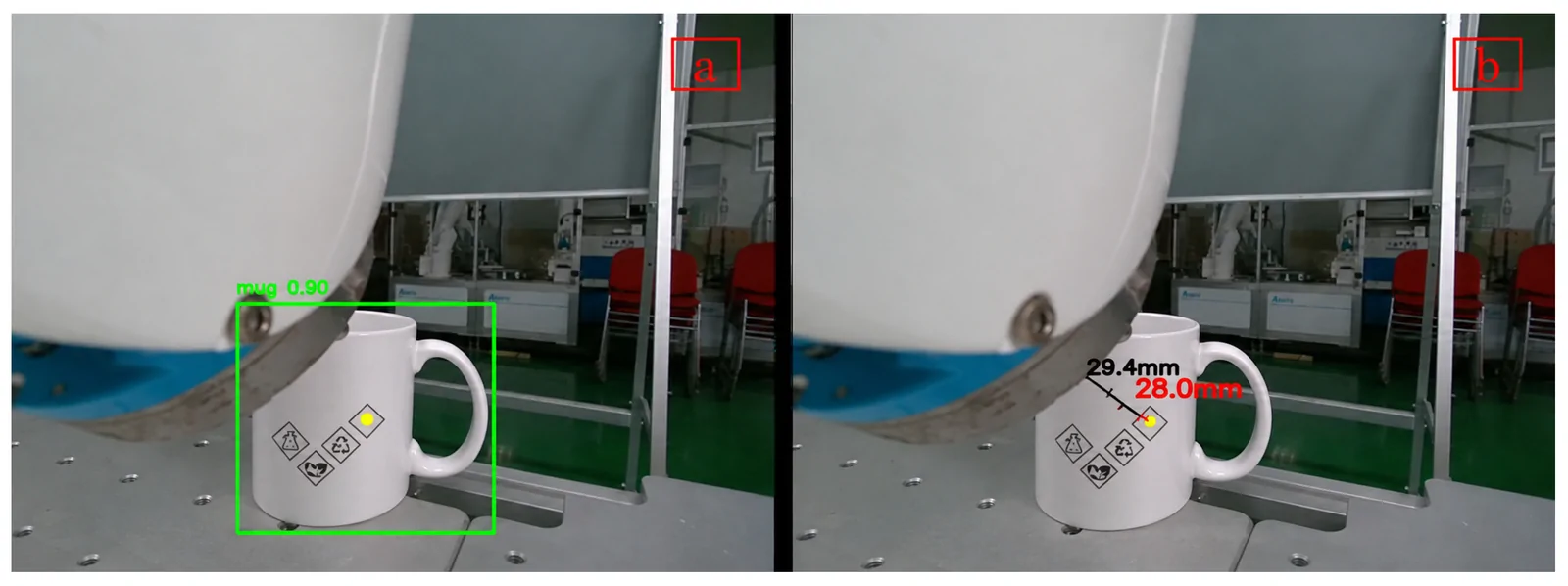
Sixteenth experiment: (**a**) YOLOv11 detection, (**b**) robotic arm end-effector localization error.

In addition, Figure 13 shows the final sixteenth experiment: (a) YOLOv11 detection with 0.90 confidence, and (b) the robotic arm’s end-effector. With planar and depth errors of 28.0 mm and 29.4 mm, the results show stable, reliable localization, maintaining accuracy and robustness despite slightly lower detection confidence.

Among all experiments, experiment 4 achieved the best performance with the lowest planar and depth errors, indicating optimal alignment between visual detection and robotic position. Errors remained stable across all trials, within ±3 mm, proving consistent and reliable performance. The results of all 16 trials are summarized in Table 2. For each trial, the planar positioning error was calculated as the Euclidean distance between the robotic-arm endpoint coordinate and the detected target-center coordinate in the X–Y plane. The Z error represents the absolute difference between the corresponding depth-direction coordinates. These quantities therefore characterize the coordinate discrepancy between the vision-derived target position and the robotic-arm reference used in the experiment. The target coordinates in the present experiments were not independently validated using an external metrology-grade ground-truth system. The reported errors were determined from the coordinate differences between the vision-derived target position and the robotic-arm/end-effector reference used in the experimental setup. Therefore, these measurements quantify the integrated system-level localization discrepancy rather than the absolute accuracy of the D455 depth camera alone.

Although the variation among repeated localization experiments is relatively small, the absolute positioning deviation remains substantial for fine maintenance operations. The average planar and depth errors are approximately 27.7 mm and 30.1 mm, respectively. Examination of the signed coordinate differences indicates a systematic rather than purely random offset. Across the 16 trials, the robotic-arm *X* coordinate was consistently lower than the detected target-center *X* coordinate, with a mean signed offset of approximately −21.6 mm, while the *Y* direction exhibited a mean signed offset of approximately −17.1 mm. Potential contributors include residual camera-to-robot extrinsic-calibration error, RGB-D depth uncertainty, robot/tool-frame calibration error, and displacement between the YOLO bounding-box center and the actual desired physical interaction point. Because these error sources were not independently isolated, their individual quantitative contributions cannot be determined from the present experiments.

Accordingly, the present YOLO11–RGB-D localization pipeline should be interpreted as a coarse global positioning stage rather than a final fine-alignment method for screw engagement. For practical screw-removal operations, a hierarchical coarse-to-fine strategy is required. The current vision system can first guide the manipulator to a safe pre-contact pose near the maintenance target, after which a secondary alignment method, such as image-based visual servoing, local point-cloud registration, or force/compliance-assisted alignment, can refine the end-effector pose before tool engagement. Such a secondary localization stage is necessary to reduce the current centimeter-scale error to the millimeter or sub-millimeter range required for screw manipulation. Therefore, the localization accuracy demonstrated in the present experiments should not be interpreted as sufficient for direct tool-to-screw engagement. Its present role is to bring the manipulator into a safe neighborhood of the maintenance target, after which a local fine-alignment stage would be required.

### 4.3. Analysis of Digital Twin Path in the Real Environment

**Figure 14:**
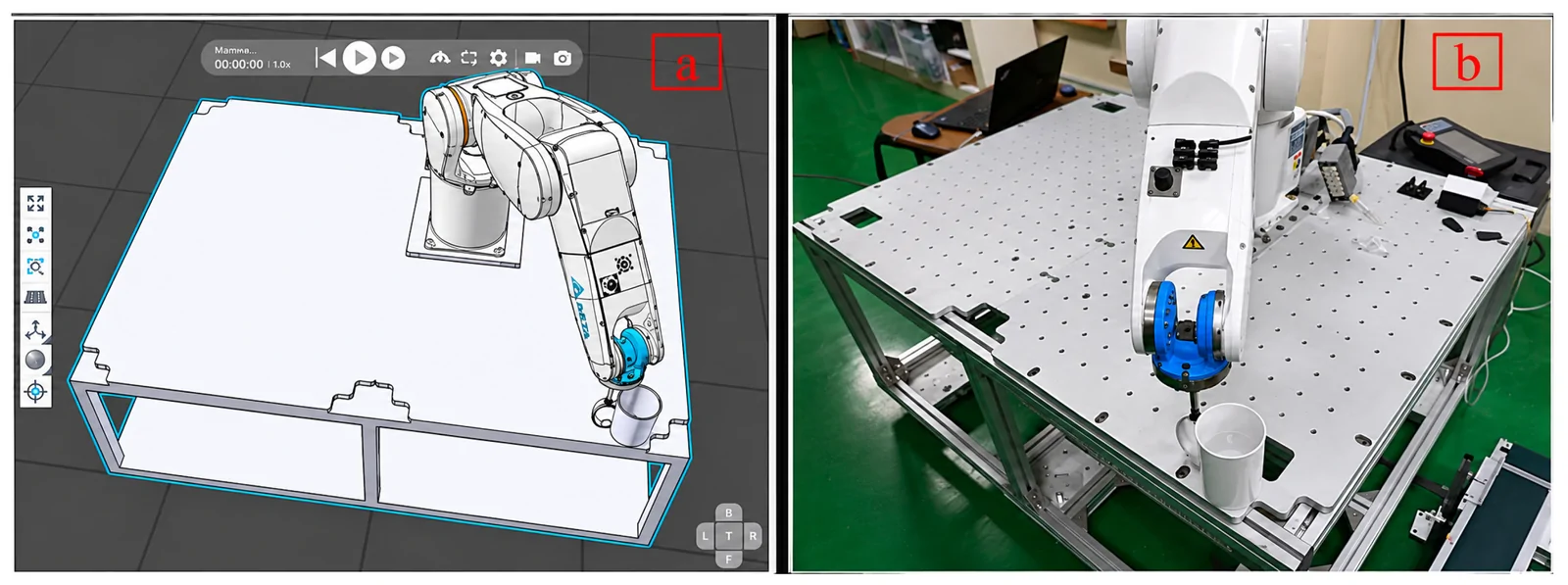
First experiment: (**a**) Path planning result in the virtual environment, (**b**) Actual robotic arm execution result.

In this study, five experiments were conducted at different workspace locations with varied turning configurations, a total of ten trials, to evaluate the application of digital-twin trajectories on the physical robotic arm. For the first test, the mug was placed 530 mm from the table’s midpoint with its handle parallel to the edge. As shown in Figure 14, the trajectory generated in the digital-twin environment was transferred to the physical robotic arm, which successfully reached the designated target region. This experiment therefore demonstrates trajectory-transfer feasibility.

**Figure 15:**
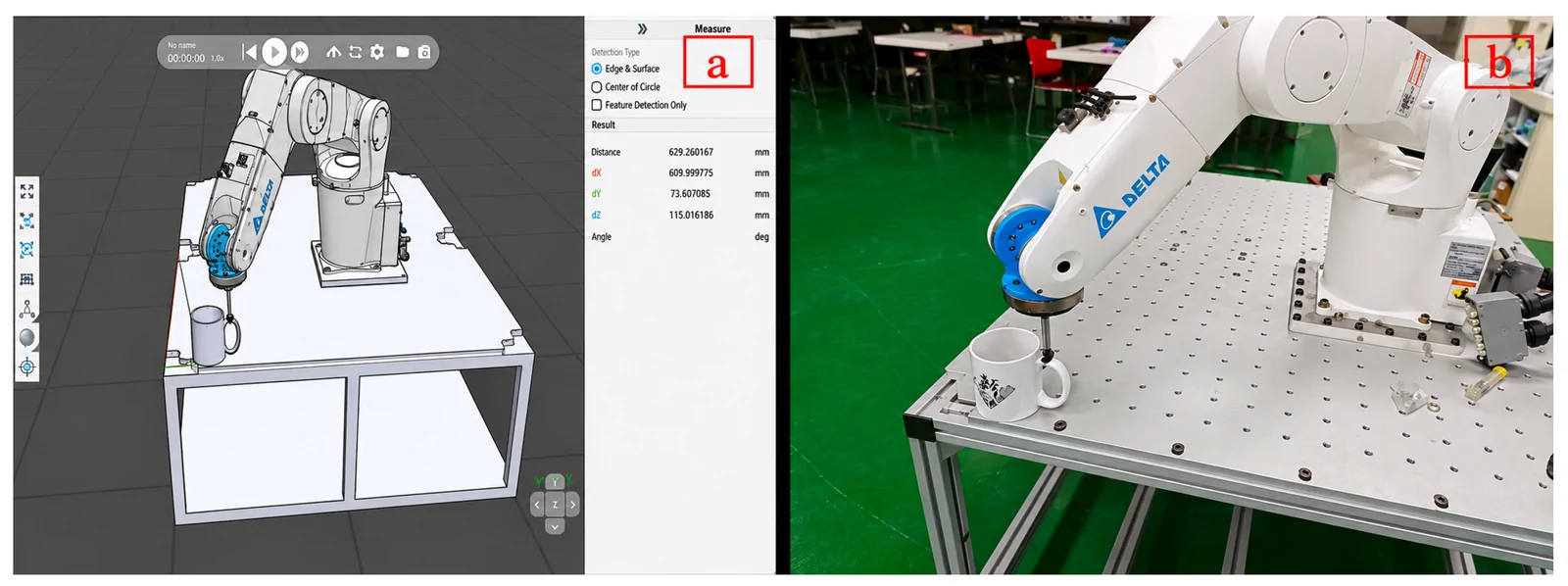
Third experiment: (**a**) Object placement and path planning in the virtual environment, (**b**) Actual robotic arm execution.

Moreover, in the third setup, the mug was positioned aligned with the short edge of the table, at a distance of x = 610 mm and y = 75 mm from the midpoint of the table’s long edge, with the handle oriented parallel to the short edge. The virtual trajectory was successfully transferred to the physical robotic arm, and the arm reached the designated target region, as shown in Figure 15.

**Figure 16:**
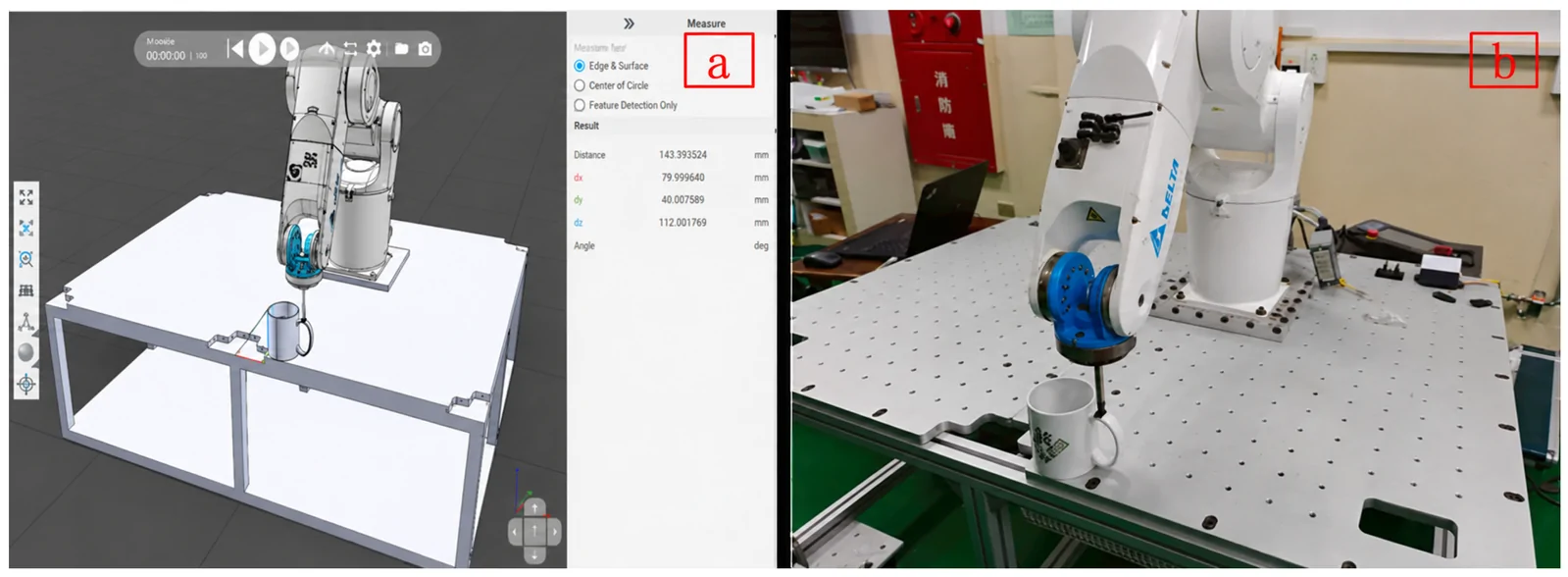
Fifth experiment: (**a**) Object placement and path planning in the virtual environment, (**b**) Actual robotic arm execution result.

In the fifth setup, the mug was positioned aligned with the long edge of the table, at a distance of x = 80 mm and y = 40 mm from the midpoint of the table’s long side, with the handle oriented parallel to the long edge. The virtual path generated by the digital twin was consistent with the actual trajectory executed by the robotic arm, accurately reaching the target position, as shown in Figure 16.

**Figure 17:**
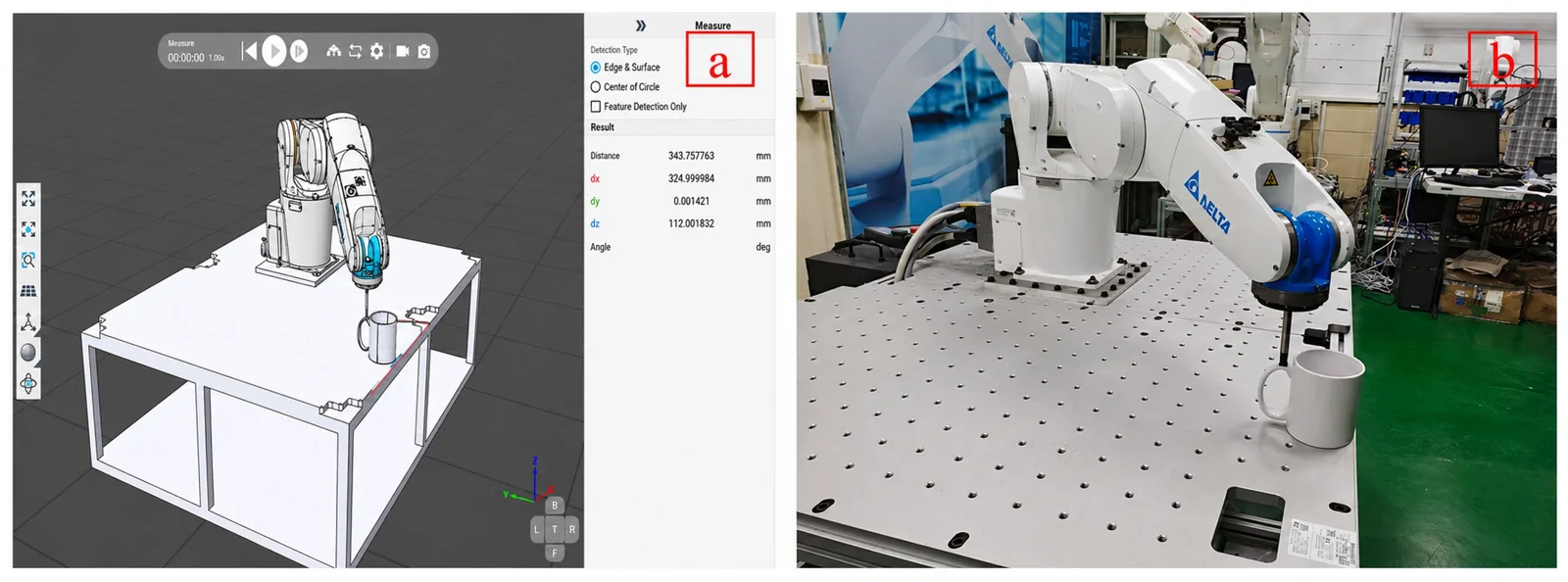
Ninth experiment: (**a**) Object placement and path planning in the virtual environment, (**b**) Actual robotic arm execution.

In the ninth setup, the mug was positioned aligned with the long edge of the table, at a distance of x = 325 mm from the midpoint of the table’s long side, with the handle oriented perpendicular to the long edge. The virtual path planning results were consistent with the actual execution, and the robotic arm accurately reached the target position, as shown in Figure 17.

**Figure 18:**
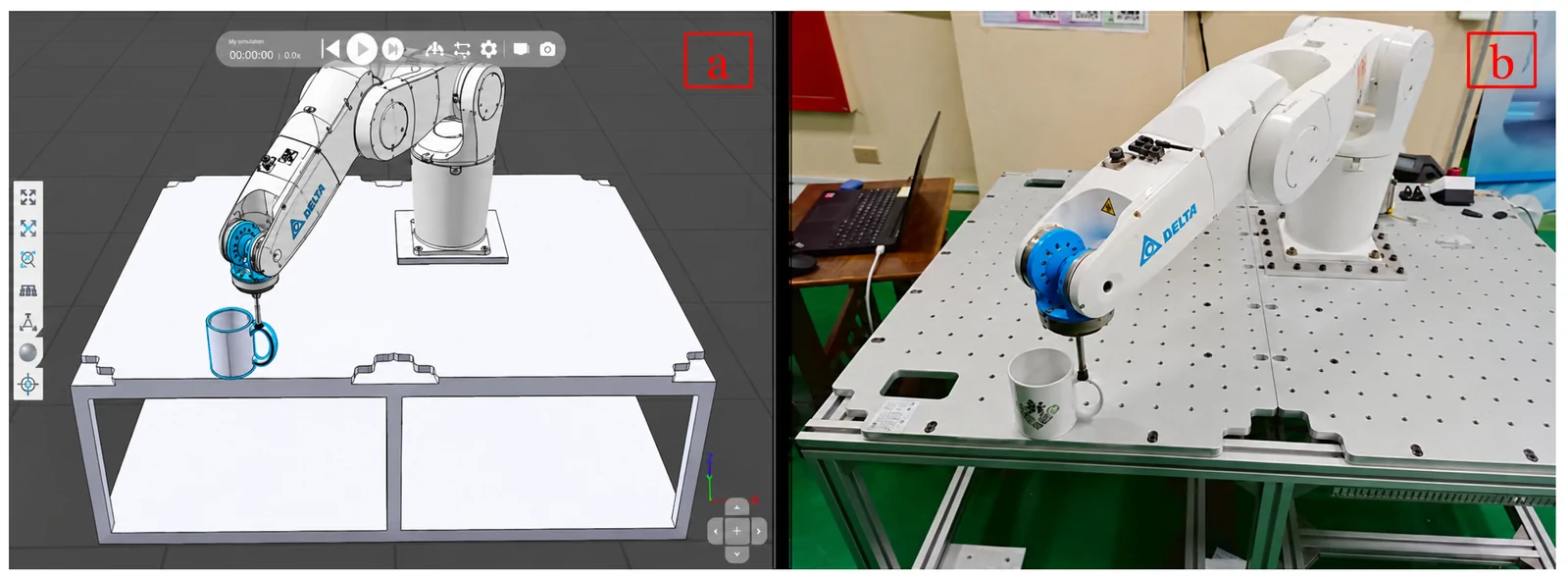
Tenth experiment: (**a**) Path planning result in the virtual environment, (**b**) Actual robotic arm execution.

Finally, in the tenth experimental setup, the mug’s handle was rotated to be parallel with the table’s long edge. As shown in Figure 18, the digital-twin-generated trajectory was successfully executed by the physical robotic arm, which reached the designated target region. Figure 18a shows the path-planning result in the virtual environment, and Figure 18b shows the actual robotic arm execution.

All ten simulated maintenance trajectories successfully reached their designated maintenance targets, corresponding to a reachability rate of 100% (10/10). No collision events were detected during the evaluated trajectories, corresponding to a collision occurrence rate of 0% (0/10) and a collision-free trajectory rate of 100% (10/10). These results quantitatively demonstrate reachability and collision feasibility under the evaluated digital-twin configurations. This result represents a quantitative reachability outcome rather than a quantitative comparison of simulated and physical trajectory fidelity. Table 3 summarizes the reference points and digital-twin test results for the mug at different positions and orientations on the table. It provides a feasibility assessment indicating whether the planned target was successfully reached. Quantitative end-effector position and orientation deviations were not recorded during these initial trajectory-transfer trials and therefore cannot be retrospectively reported. The present results should consequently be interpreted as evidence of successful trajectory transfer and target reachability rather than quantitative trajectory fidelity. Future experiments will record commanded and measured six-degree-of-freedom end-effector poses to quantify virtual-to-physical trajectory fidelity.

**Figure 19:**
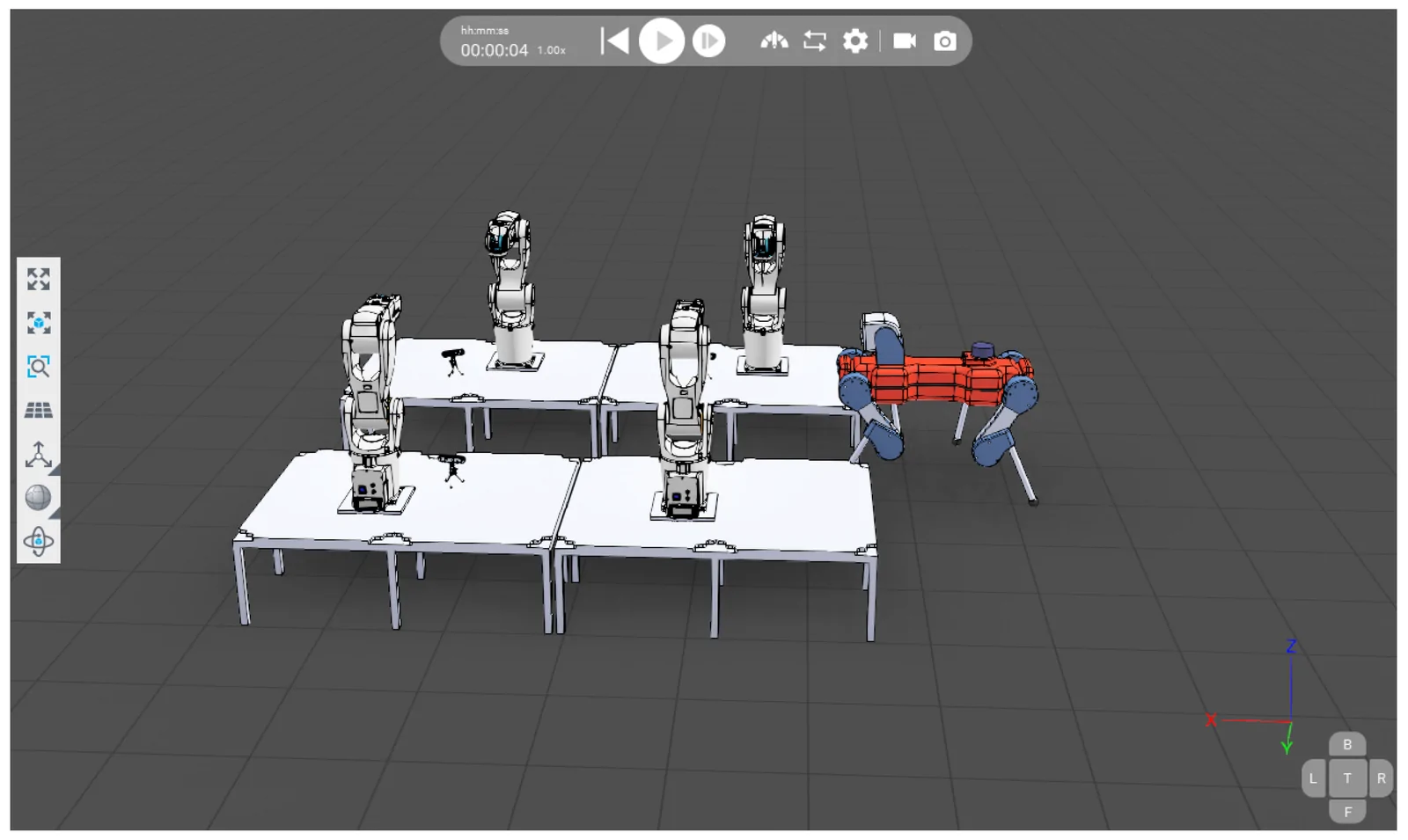
Quadruped robot moving to the designated position.

**Figure 20:**
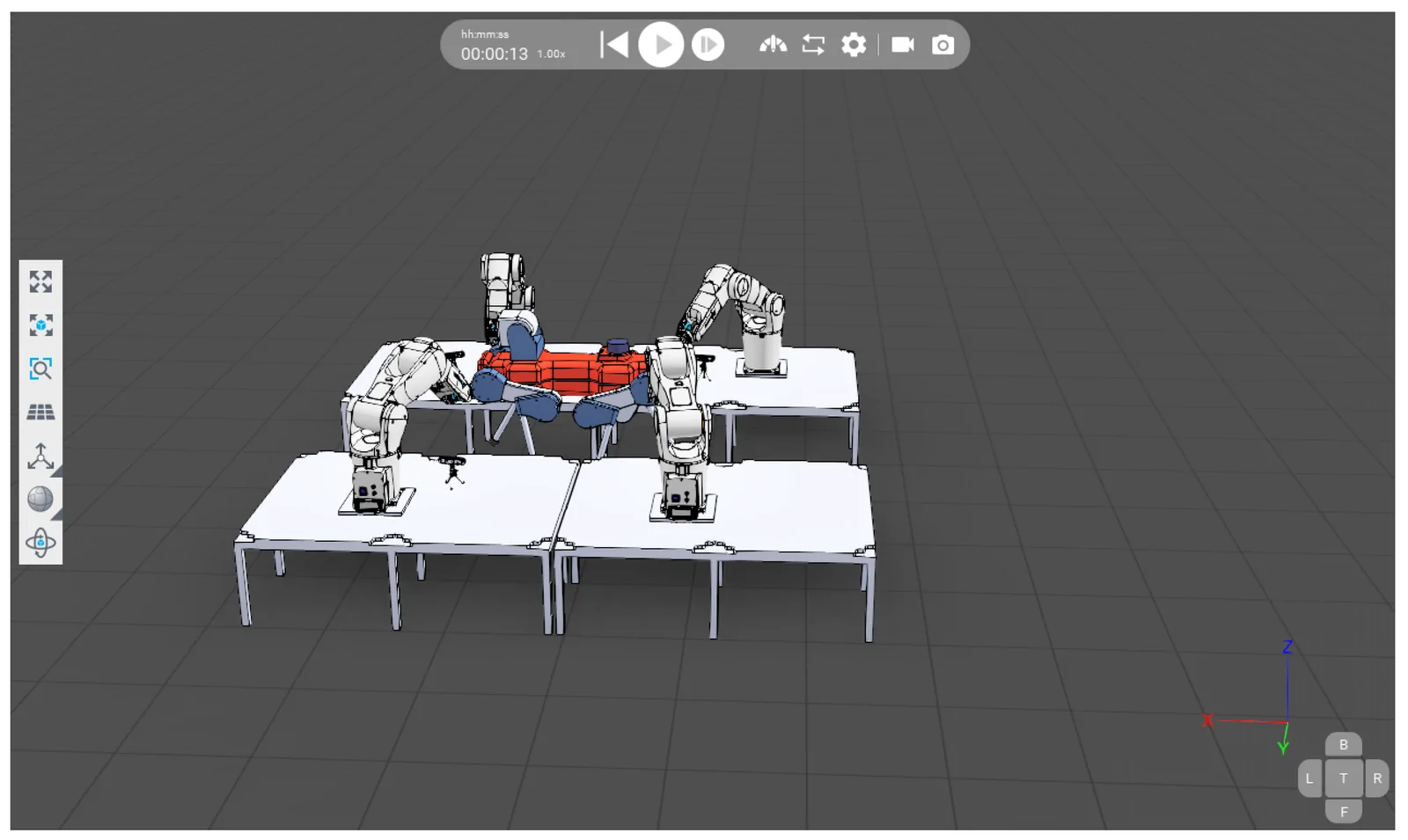
Robotic arm removes screws after the quadruped reaches position.

Following the physical proxy-object experiments, the validated workflow was extended to a simulation-based quadruped-robot maintenance scenario. The quadruped robot was first positioned within the virtual maintenance area, as shown in Figure 19. After reaching the designated virtual location, the robotic arm performed a simulated screw-removal sequence, as shown in Figure 20. The objective of this stage was to evaluate manipulator reachability, end-effector approach orientation, trajectory feasibility, and collision-free motion around the quadruped chassis. Unlike the mug experiments, the screw-removal operation shown in Figure 19 and Figure 20 was conducted only in the digital-twin environment; physical autonomous screw removal from the quadruped chassis was not performed in the present study. Accordingly, the quadruped-maintenance experiment evaluates task-level feasibility in simulation, including manipulator reachability, end-effector approach orientation, trajectory planning, and collision avoidance, rather than demonstrating complete physical autonomous maintenance. Therefore, this experiment should be interpreted as simulation-based pre-deployment validation of the maintenance workflow rather than experimental verification of a fully autonomous physical maintenance task.

**Figure 21:**
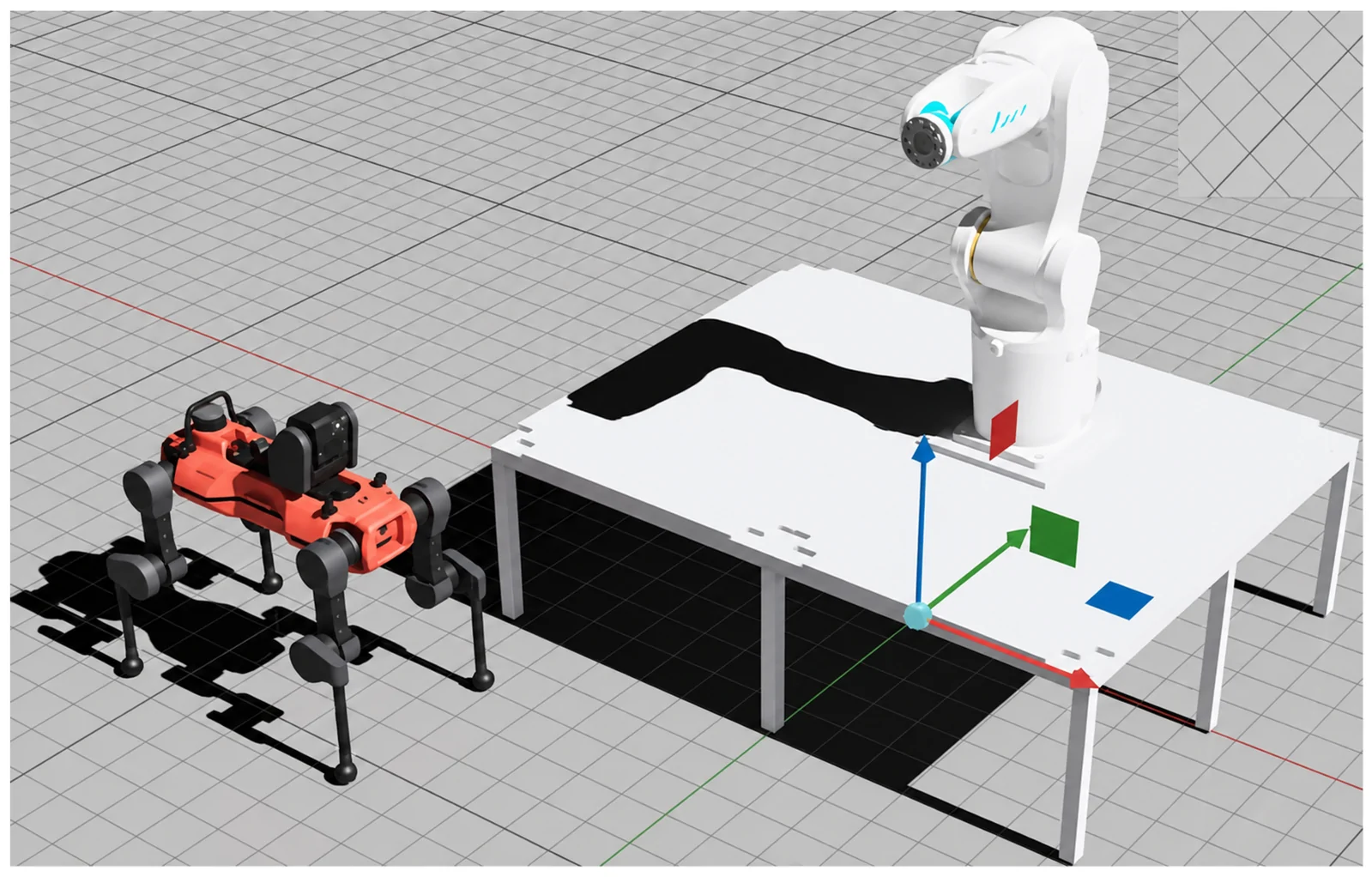
NVIDIA Omniverse visualization of the URDF model exported from DIATwin for digital twin simulation and verification of robotic maintenance tasks.

Figure 21 shows the robotic maintenance digital twin in NVIDIA Omniverse, created by importing the URDF model from DIATwin. The environment integrates the quadruped robot and 6-DOF robotic arm while preserving kinematics, joint constraints, coordinate frames, and collision geometry. Omniverse was used for workspace visualization, reachability inspection, collision checking, and physics-based pre-deployment verification under RTX simulation. This configuration enables evaluation of spatial relationships and task feasibility before physical deployment, as shown in Figure 21.

### 4.4. Secure Communication Performance Evaluation

To evaluate the feasibility of the proposed secure communication architecture, an MQTT-based secure communication link was implemented using a Raspberry Pi 4 as the intermediary gateway. TLS encryption, together with authentication and certificate-based identity verification, was applied to the MQTT communication channel. The objective of this experiment was to evaluate the feasibility of authenticated and encrypted MQTT communication within the proposed robotic architecture. The experiment was designed as a communication-feasibility assessment rather than a comprehensive cybersecurity or statistical network-performance benchmark. A total of 100 message-transfer trials were performed for each configuration, resulting in 200 sample measurements. Table 4 presents the experimental configuration, sample count, and statistical latency results for the non-TLS baseline and TLS-protected MQTT communication. The resulting latency distribution was characterized using the mean, standard deviation, minimum, and 95th-percentile latency. As shown in Table 4, the MQTT communication without TLS exhibited a mean latency of 42.8 ms with a standard deviation of 4.7 ms. The minimum latency was 34.6 ms, and the 95th-percentile latency was 50.9 ms. When TLS protection and certificate-based authentication were enabled, the mean latency was 49.6 ms with a standard deviation of 5.9 ms. The corresponding minimum and 95th-percentile latencies were 39.8 ms and 60.8 ms, respectively.

**Figure 22:**
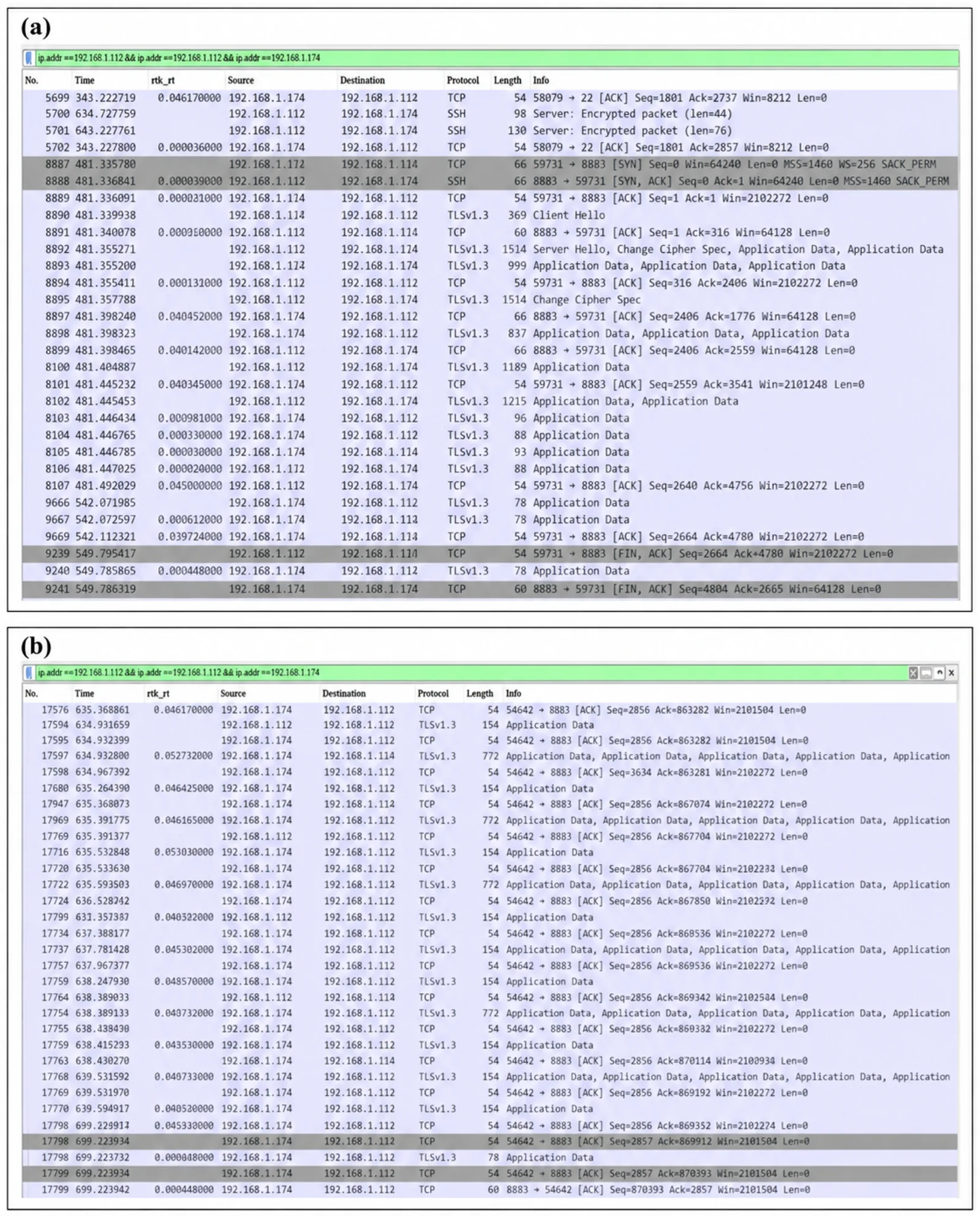
(**a**) TLS/SSL success sample, (**b**) TLS/SSL success end.

Moreover, successful TLS communication was verified through packet-level network traces. Figure 22 shows the establishment of the TLS session and subsequent encrypted application-data exchange. As shown in Figure 22a, the captured packets include the TLS handshake sequence, including the Client Hello and Server Hello messages, followed by encrypted application-data exchange. Figure 22b shows the subsequent protected communication session, in which application payloads continue to be transmitted through the TLS channel. These traces confirm successful establishment of the TLS-protected MQTT connection and encrypted application-data transfer between the communicating endpoints. The packet captures confirm secure session establishment and encrypted communication but do not demonstrate resistance to adversarial attacks. Table 4 presents the main experimental configuration and observed communication results. The secure communication test was implemented using a Raspberry Pi 4 gateway with MQTT, TLS encryption, and certificate-based authentication.

The results indicate that the proposed secure communication layer is feasible for high-level robotic command and telemetry exchange under the evaluated configuration. Although TLS encryption and certificate-based authentication were successfully established without excessive communication delay, the present experiment focused primarily on communication feasibility and latency. Throughput under sustained load, packet-loss behavior, and resilience against active cyberattacks were not quantitatively evaluated and therefore remain subjects for future investigation.

Table 5 compares the detection performance of the proposed YOLOv11-based method with recent real-time object detectors and application-specific YOLO models. In the present experimental dataset, YOLOv11 achieved an mAP@0.5 of 99.5% and an mAP@0.5:0.95 of 83.7%, improving upon YOLOv10, which achieved 98.6 and 82.2%, respectively. Application-specific studies using RSF-YOLOv8 and YOLOv8n also reported high mAP@0.5 values of 99.39% and 99.5%, respectively; their corresponding mAP@0.5:0.95 values were 71.25% and 59.76%. In comparison, general-purpose detectors evaluated on the COCO benchmark, including D-FINE and RF-DETR, reported mAP@0.5:0.95 values of 52.3% and 54.7%, respectively.

## 5. Conclusions

In this study, we developed an integrated vision-guided robotic-arm and digital-twin framework for pre-deployment validation of future quadruped-robot maintenance operations. The system first identifies the target object in the physical environment using the YOLO11 object recognition algorithm. Subsequently, external calibration between the camera and robotic arm is performed to transform image coordinates into the arm’s coordinate system. Joint angles are then calculated through inverse kinematics to guide the arm in completing positioning and task execution. The system encompasses coordinate transformation, joint angle computation, and motion control, while the digital twin simulation supports pre-deployment evaluation of task feasibility.

Moreover, the physical localization experiments demonstrated repeatable coarse target localization, with average planar and depth deviations of 27.7 mm and 30.1 mm, respectively. Although the relatively small trial-to-trial variation indicates repeatability, the centimeter-scale absolute error remains too large for direct screw engagement. Accordingly, the current YOLO11–RGB-D localization stage should be interpreted as a coarse pre-positioning method, and practical screw manipulation will require an additional fine-alignment mechanism before tool engagement. Furthermore, to evaluate the effectiveness of virtual simulation, ten positioning experiments were conducted using a mug. The arm’s movement from the initial position to the designated target was simulated in the virtual environment, and the resulting trajectory data was applied to the physical robotic arm to verify control feasibility. All ten trials successfully reached their designated targets, corresponding to a reachability rate of 100% (10/10). No collision events were detected during the evaluated trajectories, corresponding to a collision occurrence rate of 0% (0/10) and a collision-free trajectory rate of 100% (10/10). These results quantitatively demonstrate reachability and collision feasibility under the evaluated digital-twin configurations. This result demonstrates the feasibility of transferring digital-twin-generated trajectories to the physical robotic arm, demonstrating the practical value of the virtual environment. In addition, the workflow was extended to a quadruped-robot maintenance scenario, in which screw-access reachability, end-effector approach orientation, trajectory feasibility, and collision avoidance were evaluated. These results demonstrate the system’s flexibility and the digital twin framework’s potential to handle diverse, complex, and multi-step robotic tasks with high reliability. In addition, the secure communication layer was evaluated using an MQTT gateway implemented on a Raspberry Pi 4 with TLS and certificate-based authentication. Successful TLS session establishment and encrypted application-data exchange were confirmed through packet-level analysis. The TLS-protected configuration showed a mean latency of 49.6 ms and a 95th-percentile latency of 60.8 ms. These results demonstrate the feasibility of the proposed security layer for high-level command and telemetry communication, although throughput, packet-loss behavior, and active attack resilience require further quantitative evaluation. Moreover, experimental results show that our proposed YOLO11 algorithm achieves a mAP of 99.5%, providing higher object detection accuracy and significantly reduced computational time compared to YOLO10 when tested on the same environment and dataset. This demonstrates its superior performance for precise and efficient robotic maintenance tasks. Therefore, the proposed framework demonstrates the feasibility of integrating vision-guided manipulation, secure communication, and digital-twin-based pre-deployment verification for future smart maintenance and cyber–physical robotic systems.

## Figures and Tables

**Table 1:** Performance comparison of YOLO models.

Model	Precision	F1-Score	mAP@0.5	mAP@0.5:0.95	Inference Time (ms)	FPS
YOLOv10n	1.000	0.930	0.986	0.822	10.52	95.1
YOLO11n	1.000	1.000	0.995	0.837	8.47	118.0

**Table 2:** Robotic arm positioning results for 16 experiments.

Test	Arm X	Arm Y	Obj. Center X	Obj. Center Y	X–Y Error (mm)	Z Error (mm)
1	236	323	259	338	27.46	30.5
2	396	366	418	382	27.20	31.2
3	58	298	84	314	30.53	31.4
4	257	357	275	374	24.76	29.8
5	58	305	81	320	27.46	30.3
6	132	305	154	323	28.43	30.5
7	183	315	207	330	28.30	29.9
8	225	314	242	333	25.50	29.6
9	230	310	250	329	27.59	29.4
10	256	319	280	335	28.84	29.7
11	296	327	318	344	27.80	29.9
12	69	309	93	323	27.78	30.4
13	95	319	113	340	27.66	29.9
14	166	331	183	353	27.80	29.5
15	230	344	252	361	27.80	29.7
16	274	356	297	372	28.02	29.4

**Table 3:** Digital twin path test results.

Test	Reference Point Setting	Target Reached	Reachability Result	Collision Events	Collision Occurrence Rate	Collision-Free Result
1	x=530 mm	Yes	1/1 (100%)	0/1	0%	1/1 (100%)
2	x=530 mm	Yes	1/1 (100%)	0/1	0%	1/1 (100%)
3	x=610 mm, y=75 mm	Yes	1/1 (100%)	0/1	0%	1/1 (100%)
4	x=610 mm, y=76 mm	Yes	1/1 (100%)	0/1	0%	1/1 (100%)
5	x=80 mm, y=40 mm	Yes	1/1 (100%)	0/1	0%	1/1 (100%)
6	x=80 mm, y=40 mm	Yes	1/1 (100%)	0/1	0%	1/1 (100%)
7	x=80 mm, y=40 mm	Yes	1/1 (100%)	0/1	0%	1/1 (100%)
8	x=80 mm, y=40 mm	Yes	1/1 (100%)	0/1	0%	1/1 (100%)
9	x=325 mm	Yes	1/1 (100%)	0/1	0%	1/1 (100%)
10	x=325 mm	Yes	1/1 (100%)	0/1	0%	1/1 (100%)
Overall	–	10/10	10/10	0/10	–	10/10
Rate	–	100%	100%	0%	0%	100%

**Table 4:** Secure communication test configuration and results.

Parameter	Baseline	MQTT with TLS
Gateway platform	Raspberry Pi 4	Raspberry Pi 4
Communication protocol	MQTT	MQTT
Transport security	–	TLS
Authentication mechanism	–	Certificate-based authentication
Number of samples	100	100
Mean latency (ms)	42.8	49.6
Standard deviation (ms)	4.7	5.9
Minimum latency (ms)	34.6	39.8
95th-percentile latency (ms)	50.9	60.8
TLS session establishment	–	Successful
Encrypted application-data transfer	–	Successful

**Table 5:** Comparison of object detection methods and detection performance.

Method and Reference	Dataset	mAP@0.5	mAP@0.5:0.95
D-FINE [31]	COCO val2017	–	52.3
RF-DETR [32]	COCO	73.6	54.7
RSF-YOLOv8 [33]	Experimental	99.39	71.25
YOLOv8n [34]	Experimental	99.5	59.76
YOLOv10 (ours)	Experimental	98.6	82.2
YOLOv11 (ours)	Experimental	99.5	83.7

## Data Availability

The data presented in this study are available in this article.
